# Single‐Cell Annotation and Localization via Integrating Spatial Transcriptomics Maps the Mouse Ocular Atlas and RAO Dynamics

**DOI:** 10.1002/advs.75857

**Published:** 2026-06-01

**Authors:** Chen Du, Yinming Li, Ziyue Li, Yuedan Wang, Shaopeng Li, Yijie Wang, Yingying Qu, Bingyang Lv, Ying Li, Ting Chen, Yu Zhou, Xuan Xiao

**Affiliations:** ^1^ College of Life Sciences TaiKang Center for Life and Medical Sciences Department of Ophthalmology Renmin Hospital of Wuhan University Wuhan University Wuhan Hubei China; ^2^ Department of Clinical Laboratory Institute of Translational Medicine Renmin Hospital of Wuhan University Wuhan Hubei China; ^3^ Frontier Science Center for Immunology and Metabolism Wuhan University Wuhan Hubei China

## Abstract

Retinal artery occlusion (RAO), a blinding emergency, demands clarification of cellular spatiotemporal dynamics for targeted therapies. Single‐cell RNA‐seq is powerful but lacks spatial context, whereas spatial transcriptomics struggles with cell segmentation errors and low transcript capture efficiency. To overcome these, we developed the ASCAL (Automated Single‐Cell Annotation and Localization) pipeline, integrating complementary spatial transcriptomics techniques: single‐nucleus‐resolution SeekSpace for cell annotation reference and uniform‐coverage Stereo‐seq for localization of annotated cells. This strategy enables automated annotation and localization of large‐scale scRNA‐seq datasets with minimal spatial sections. Leveraging ASCAL, we constructed a mouse whole‐eye single‐cell spatial atlas precisely mapping cell types across the ciliary body and retina. Moreover, we revealed pronounced spatial immune activation specifically enriched in the ganglion cell layer (GCL) in the RAO model, further validated by immunofluorescence staining. We also uncovered a selective depletion of active Rods. Importantly, RNAscope assays independently validated both the peripheral localization of this active Rod subcluster and its pronounced loss following RAO. Overall, this study provides a comprehensive spatial cell atlas of the whole eye and offers novel spatial‐resolution insights into the pathological mechanisms of RAO, serving as a valuable resource for deciphering the physiological and pathological landscapes of the eye at single‐cell resolution.

## Graphical abstract

We developed the ASCAL pipeline, integrating complementary spatial transcriptomics, to construct a high‐fidelity mouse whole‐eye single‐cell atlas. Applying ASCAL to a retinal artery occlusion (RAO) model revealed spatially restricted immune activation localized to the ganglion cell layer and the selective depletion of a translationally active, outer‐layer Rod subcluster, uncovering compartmentalized ischemic pathologies.

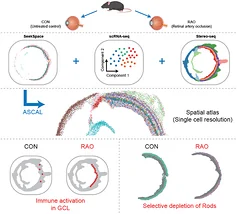

## 1 Introduction

Precise characterization of cellular heterogeneity and spatial organization is fundamental to understanding tissue development [1, 2, 3], homeostasis [4, 5, 6], and disease pathogenesis [7, 8, 9, 10]. Although single‐cell RNA sequencing (scRNA‐seq) has revolutionized the identification of diverse cell types across organs, its lack of spatial context hinders the accurate annotation of cell types and the interpretation of their functions, especially in complex or poorly characterized tissues. Spatial transcriptomic (ST) technologies have emerged to address this gap by capturing transcriptomes within their native tissue architecture, thereby enhancing the annotation and integration of cell types and functions [11, 12]. However, current ST approaches, especially high‐resolution platforms, which often rely on computational image segmentation, face significant challenges, including inaccurate cellular boundary identification, false capture of transcripts from multiple cells, low transcript counts per spot, and sparse gene detection, especially in densely packed and morphologically diverse tissues [13]. Moreover, the high cost of ST technologies further constrains the feasibility of resolving these resolution and sensitivity limitations through large‐scale experiments.

To leverage the strengths of both scRNA‐seq and ST, various computational approaches have been developed to integrate these datasets. However, a prevailing paradigm in current reference‐based integration workflows is the heavy reliance on scRNA‐seq datasets as the primary reference to annotate or deconvolute spatial transcriptomics data. Conventional scRNA‐seq annotation relies predominantly on manual curation, guided by presumed expression patterns of marker genes. The absence of spatial context compromises the classification accuracy, and subjective interpretation of marker gene expression patterns may introduce bias, especially in ambiguous expression patterns. Computational methods, such as reference‐based tools like SingleR [14] and CellTypist [15], utilize bulk RNA‐seq or scRNA‐seq atlases for automated labeling but encounter limitations due to incomplete reference datasets and technical batch effects. More recently, foundation models such as scGPT [16], pre‐trained on extensive scRNA‐seq datasets exceeding 30 million cells, show promise but exhibit constrained zero‐shot performance due to the scope limitations of the pre‐training data [17]. Consequently, reliably identifying cell types without strong prior knowledge or subpopulations lacking definitive markers remains a significant challenge. Since spatial context provides independent biological evidence for cell identity, we posit that integrating spatial data could overcome these limitations. However, there is no method that can annotate single‐cell data based on spatial information to cost‐effectively construct a high‐confidence, high‐throughput, and spatially resolved single‐cell atlas.

**FIGURE 1:**
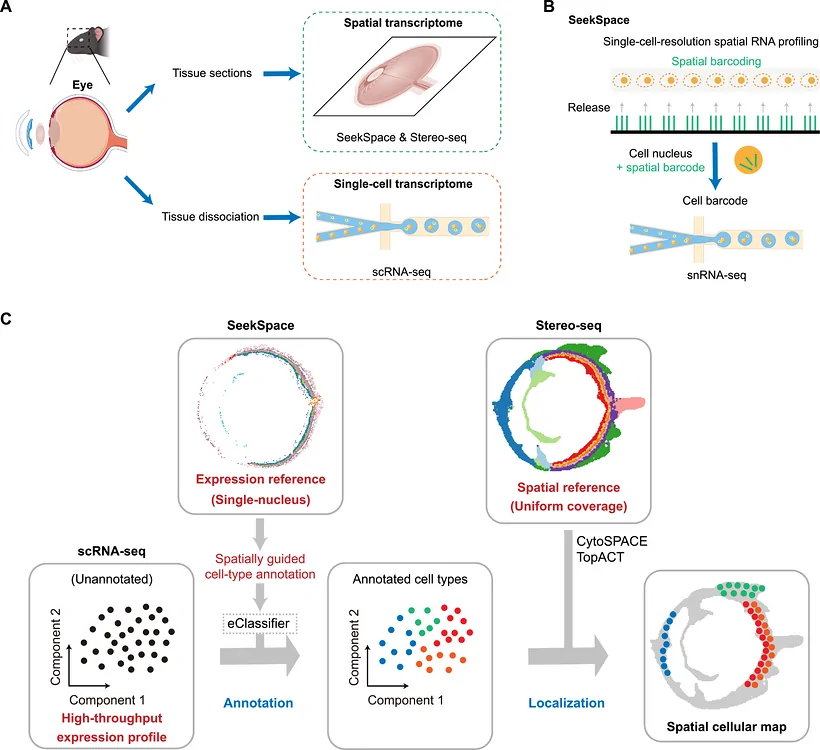
Overview of single‐cell resolution spatial transcriptome profiling of the mouse eye. (A) Illustration of the experimental design. Spatial transcriptomics were performed on tissue sections of mouse eyes, and scRNA‐seq was conducted following single‐cell dissociation of mouse eye tissues. (B) Illustration of SeekSpace workflow for spatial transcriptome. The spatial barcodes are released into the cell nucleus in situ, followed by snRNA‐seq with cell barcodes. (C) Overview of the strategy ASCAL (Automated Single‐Cell Annotation and Localization) for cell annotation and localization by integrating multimodal data. SeekSpace data provides an expression reference with spatially guided cell‐type annotation for training eClassifier. eClassifier is applied to individual cellular expression profiles from high‐throughput scRNA‐seq data to assign cell types. The uniform‐coverage Stereo‐seq data guides the spatial localization of annotated single cells.

Here, we developed ASCAL (Automated Single‐Cell Annotation and Localization), a pipeline capable of automatically annotating and localizing single‐cell data by integrating two complementary spatial transcriptomics technologies. At its core, ASCAL constructs a high‐confidence reference atlas by integrating known marker gene expression with anatomical localization of cells derived from SeekSpace, an image segmentation‐free, single‐nucleus spatial transcriptomics platform [18] (Figure 1A,B). This atlas serves as a standardized reference enabling robust and unbiased automatic annotation of scRNA‐seq datasets derived from homologous tissues. Moreover, ASCAL incorporates high‐tissue‐coverage Stereo‐seq data, and utilizes CytoSPACE [19] and TopACT [20] algorithms to achieve precise spatial mapping of both major and rare cell types within single‐cell datasets. By integrating high‐throughput single‐cell data, this innovative approach also effectively addresses the limitations of low cell throughput typically associated with frozen sections, thereby enhancing the overall quality and reliability of spatial transcriptomic analysis. With a limited number of tissue sections, ASCAL utilizes SeekSpace to construct a gene expression reference and Stereo‐seq to provide spatial positioning reference, enabling the accurate annotation and localization of large‐scale scRNA‐seq datasets (Figure 1C).

We validated the utility of ASCAL in complex biological systems by constructing a high‐fidelity, single‐cell spatial atlas of the mouse eye. The eye is a highly structured organ composed of distinct anatomical regions, such as the ciliary body, sclera, and lens, each with unique molecular profiles and physiological roles [21]. Beyond its structural complexity, the eye contains densely packed cells and encompasses a wide array of both neural and non‐neural cell types, resulting in extensive cellular diversity and pronounced spatial heterogeneity. These features pose significant challenges for accurate spatial mapping, and as a result, existing spatial atlases of the eye remain incomplete. Besides, previous single‐cell studies have primarily focused on the specific tissue in the eye, such as retina [22, 23, 24], ciliary body [25, 26], iris [27], cornea [28], and lens [29], but the markers of many other essential ocular compartments, like sclera, remain poorly characterized at single‐cell resolution. Our work fills this gap by providing the most comprehensive spatially resolved single‐cell atlas of the mouse eye to date, offering a valuable resource for deciphering the cellular architecture and function of this intricate organ.

Moreover, we applied ASCAL to investigate the cellular and molecular responses following RAO injury, a pathological ischemic condition affecting the retina [30, 31], by using the unilateral pterygopalatine ophthalmic artery occlusion (UPOAO) mouse model [32]. We uncovered rare cell populations that were previously missed by unsupervised clustering, and identified RAO‐induced immune activation and specific Rod subtype reduction at single‐cell resolution with precise spatial mapping through ASCAL. This delineated compartmentalized inflammation and subtype‐specific vulnerability within the retinal microenvironment under RAO, thereby offering a molecular basis for mechanistic study and potential therapeutic intervention.

## 2 Results

### 2.1 Single‐Cell Resolution Spatial Transcriptome Profiling of the Mouse Eye With SeekSpace

To establish a high‐confidence expression reference, we performed SeekSpace spatial transcriptomics on three frozen mouse eye sections (Figure 1A,B), generating a single‐cell resolution map comprising 17,835 cells. SeekSpace physically isolates single nuclei after spatial barcoding using a microfluidic barcoding platform, thereby eliminating the need for deconvolution algorithms or image‐based cell segmentation. Additionally, by employing single‐nucleus sequencing, SeekSpace facilitates the identification of a broader spectrum of cell types, including long‐axon cells. Mapping cells back to their spatial coordinates effectively reconstructs the eye's anatomical morphology while preserving its structural organization (Figure S1A). Finally, the resulting dataset exhibited a median UMI count of 413 and a median gene count of 254 per cell (Figure S1B), demonstrating its effectiveness in capturing spatially resolved cellular transcriptomic profiles.

To enhance the annotation of cell populations, we integrated marker gene expression profiles with anatomical location characteristics of various cell types. Specifically, we partitioned the cells into 37 unsupervised clusters. For glial cell and retinal cell populations, previously well‐established marker genes could be leveraged, such as *Pax6* for amacrine cells (ACs), *Vsx2* for bipolar cells (BCs), *Pde6a* for rod photoreceptors (Rods), *Slc17a6* for retinal ganglion cells (RGCs), *Onecut2* for horizontal cells (HCs), *Pde6c* for cone photoreceptors (Cones), *Vim* for Müller glia, *Mbp* for oligodendrocytes, and *Itgam* for microglia. In contrast, for the clusters in non‐retinal tissues lacking canonical marker genes, such as the lens and ciliary body, their identities were assigned based on their anatomical localization within SeekSpace coordinates, cross‐referenced with murine ocular histology.

**FIGURE 2:**
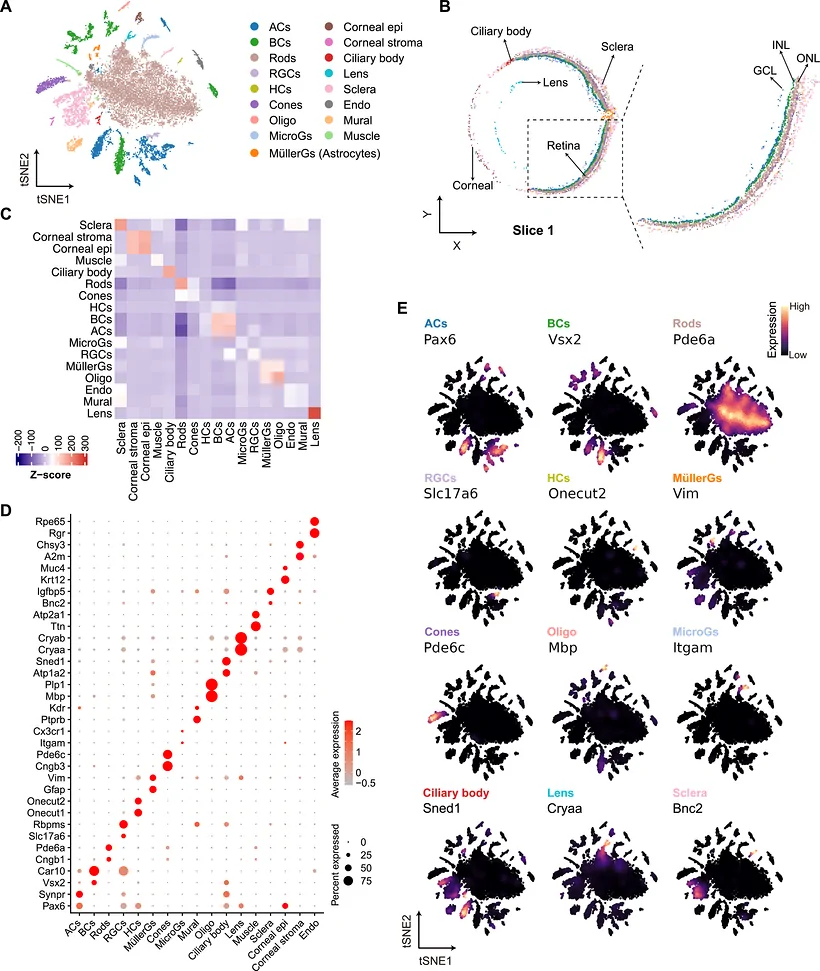
Cell type annotation of the entire mouse eye from SeekSpace data. (A) tSNE projection of annotated single‐cell clusters from SeekSpace data. (B) A tissue‐space coordinate plot of Slice 1 showing annotated cell types. Canonical anatomical structures are labeled on the plot. GCL, ganglion cell layer; INL, inner nuclear layer; ONL, outer nuclear layer. (C) Heatmap displaying the spatial neighborhood enrichment Z‐scores between transcriptomically defined cell clusters. (D) Dot plot showing the expression of representative cell‐type‐specific marker genes. Dot size and color represent expression proportion and expression level, respectively. (E) tSNE projections visualizing expression patterns of cell‐type‐specific marker genes. Color gradients indicating gene expression density distributions.

Finally, we annotated 6 retinal clusters, 3 glial clusters, and 8 other clusters on SeekSpace data (Figure 2A). These clusters exhibited strong tissue specificity and revealed well‐defined layering patterns in the retina (Figure 2B, Figure S1A). To evaluate the spatial coherence of the transcriptomic clusters, we performed a spatial neighborhood enrichment analysis. The results show that clusters exhibit significant in situ co‐localization within their physical compartments, such as the AC and BC synaptic layers, and the corneal stroma‐epithelium module, confirming that the transcriptomic clustering accurately reconstructs the anatomical lamination of the eye (Figure 2C). Cellular composition analysis revealed retinal cells as the predominant population of total cells, with Rods representing the largest subset, followed by ACs and BCs (Figure S1C,D). The distribution pattern was consistent with the known cellular composition of the mouse retina [33]. Furthermore, the specific expression of marker genes across cell populations and spatial domains further validated the reliability of our annotation (Figure 2D,E, Figure S1E,F). Additionally, by leveraging the spatial information inherent in the SeekSpace data, we identified distinct marker genes specifically associated with various non‐retinal ocular tissues. This spatially guided approach enabled the precise demarcation and molecular characterization of structures beyond the retina, providing valuable insights into their unique transcriptional signatures. In sum, a high‐confidence single‐cell resolution atlas of the mouse eye has been constructed, providing a valuable reference for the annotation of scRNA‐seq datasets from eyes.

### 2.2 Reference‐Based Automated Cell‐Type Annotation of Single Cells Across Distinct Regions of the Mouse Eye

**FIGURE 3:**
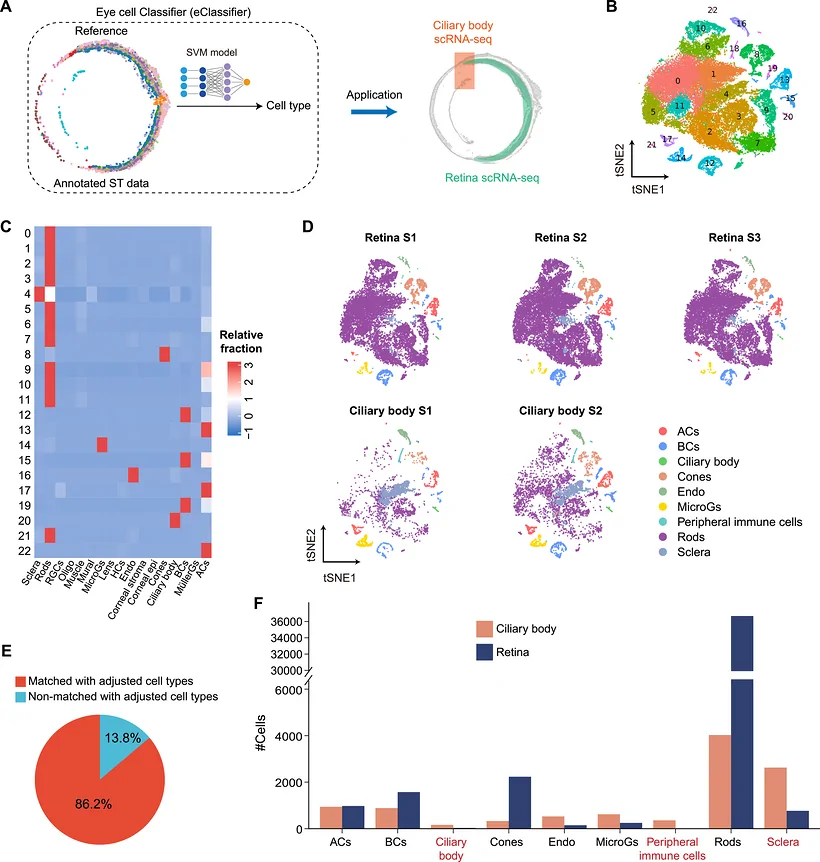
Automated cell‐type annotation based on SeekSpace reference for single cells across distinct regions of the mouse eye. (A) Schematic illustration of the automated cell‐type annotation workflow. A support vector machine (SVM) classifier was trained on the annotated SeekSpace reference atlas for cell‐type assignments of mouse eyes‐derived single‐cell data (eClassifier). (B) tSNE projection of clusters from unsupervised clustering. (C) Heatmap showing eClassifier‐predicted cell type distribution in clusters from unsupervised clustering. (D) tSNE projection of eClassifier‐annotated single‐cell clusters in each sample. (E) Pie plot showing the percentage of cells’ predicted identity matched with identity adjusted based on unsupervised clustering. (F) Bar plot showing the cell number of each cell type in the ciliary body and retina samples, respectively.

To overcome limitations of manual, marker‐dependent scRNA‐seq annotation, we implemented automated cell typing using a spatial‐informed reference atlas for mouse ocular tissues. Specifically, we constructed an eye cell classifier (eClassifier) based on a support vector machine (SVM) model, using the mouse eye single‐cell atlas generated by SeekSpace, which enables cell‐type classification of input single‐cell expression profiles. To assess the accuracy of the classification results, we performed single‐cell sequencing on isolated retinal tissues (Figure S2A) and integrated these with publicly available single‐cell data (GSE178667) from ciliary body‐associated regions [25] (Figure S2B), systematically evaluating the classifier's capability to distinguish tissue‐specific cell populations (Figure 3A).

Given the paucity of captured peripheral immune cells in tissue sections compared to their established presence in non‐retinal tissues, we first annotated peripheral immune cell populations through expression of previously validated specific marker genes (Figure S2C), followed by applying eClassifier to characterize remaining cells (Figure S2D). Then, we compared the predicted cell types of individual single cells with the results of unsupervised clustering (Figure 3B). The analysis revealed that nearly all cells within each unsupervised cluster exhibited consistent predicted labels (Figure 3C), validating the reliability of this classification approach.

Subsequently, we implemented a label reconciliation pipeline where unsupervised clustering‐derived cell populations were reannotated based on predominant eClassifier‐assigned labels (Figure 3D, Figure S2E). This cross‐validated method demonstrated high consistency, with over 86% of cellular identities maintaining identical cell‐type assignments before and after label correction (Figure 3E). Quantitative analysis of cellular distribution patterns revealed distinct tissue‐specific compartmentalization, with ciliary body, sclera, and peripheral immune cells showing significantly higher proportions in ciliary body associated tissues compared to retina, and Rods dominating in retinal samples, conversely (Figure 3F, Figure S2F). This differential cellular distribution corresponds to well‐established tissue‐specific anatomical features, thereby independently verifying the classification accuracy of the eClassifier annotation strategy.

### 2.3 A Comprehensive Transcriptome Atlas of the Entire Mouse Eye Construction Using Stereo‐seq as a Spatial Reference

**FIGURE 4:**
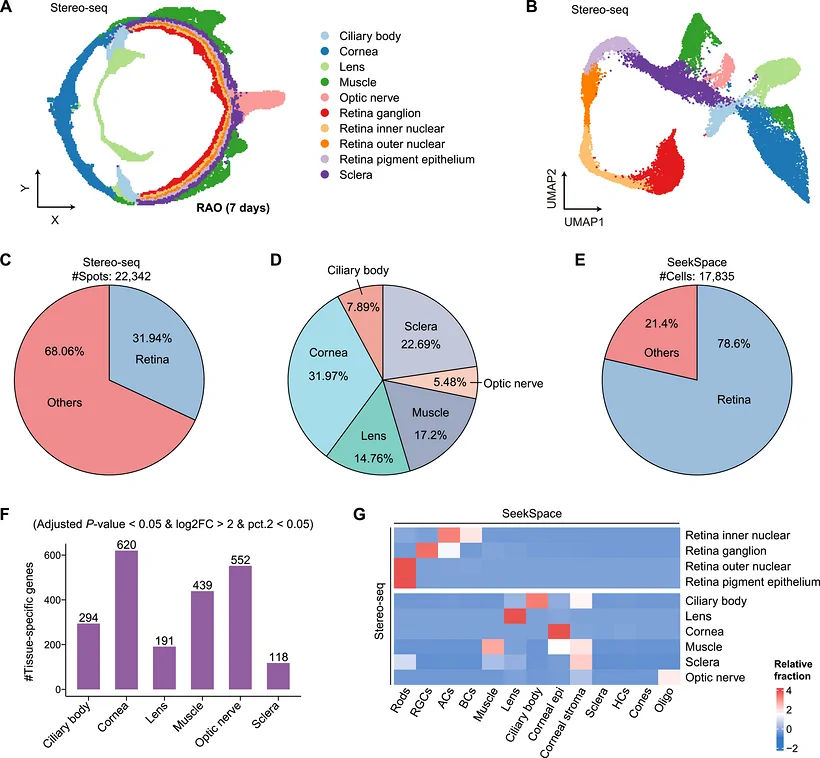
Stereo‐seq serving as a spatial reference for the entire mouse eye. (A) Space coordinate of Stereo‐seq with annotated clusters at bin50 resolution (RAO, 7 days). (B) UMAP projection of Stereo‐seq annotated clusters at bin50 resolution. (C) Pie plot showing the percentage of Stereo‐seq detected spots belonging to the retina or not. (D) Pie plot showing the percentage distribution of Stereo‐seq detected spots in each non‐retina region. (E) Pie plot showing the percentage of SeekSpace‐detected cells belonging to the retina or not. (F) Bar plot illustrating the number of tissue‐specific genes in each non‐retina region identified by Stereo‐seq at bin50. (G) Heatmap showing cell‐type deconvolution results of Stereo‐seq (bin50 resolution) based on SeekSpace. Only the cell types with specific anatomical localization are shown here for clarity.

While the SeekSpace platform achieved single‐cell resolution in spatial transcriptomic profiling, limitations associated with cryosection quality and integrity of the cell nucleus resulted in suboptimal cellular capture efficiency. To enhance the completeness of spatial reference, we implemented subcellular‐resolution Stereo‐seq on four mouse eye sections. First, we used bin50 (50 × 50 DNB bins) as a spot unit, and a median of 2,551 UMIs and 770 genes were detected per bin50 spot (Figure S3A). Clustering analysis of bin50 Stereo‐seq data revealed anatomically concordant spatial domains, with retinal structure resolving into four distinct laminae (ganglion layer, inner nuclear layer, outer nuclear layer, and pigment epithelium layer) (Figure 4A, Figure S3B), exhibiting a continuum across these retinal structures in the embedding space (Figure 4B).

Compared to SeekSpace, Stereo‐seq exhibited more uniform spatial distribution of spots, with 68% of spots localized to non‐retinal regions (Figure 4C). These non‐retinal spots showed a predominant distribution in the cornea and sclera (Figure 4D). In contrast, SeekSpace detected only 21% non‐retinal cells (Figure 4E). This enhanced coverage establishes Stereo‐seq as a critical complement for comprehensive ocular spatial atlas construction, particularly in non‐retinal regions. Furthermore, Stereo‐seq enabled the identification of tissue‐specific genes in these non‐retinal areas (Figure 4F), whose functional annotations align with the physiological roles of their corresponding anatomical structures (Figure S3C). Notably, even under a stringent fold change cutoff of 4, a substantial number of tissue‐specific genes were identified, reflecting the high transcriptional specificity of these compartments.

To resolve cellular heterogeneity within bin50 spots of Stereo‐seq, we performed deconvolution analysis using SeekSpace‐annotated cell types as a reference. Each spot was annotated by its predominant cell type, exhibiting high tissue specificity (Figure S4). Retinal spots were predominantly resolved into Rods, RGCs, ACs, and BCs, while spots in non‐retinal regions were accurately mapped to corresponding stromal and immune populations (Figure 4G). This spatial concordance validated the consistency between the two distinct spatial transcriptomic platforms.

### 2.4 Spatial Mapping of Single Cells Based on Stereo‐seq

**FIGURE 5:**
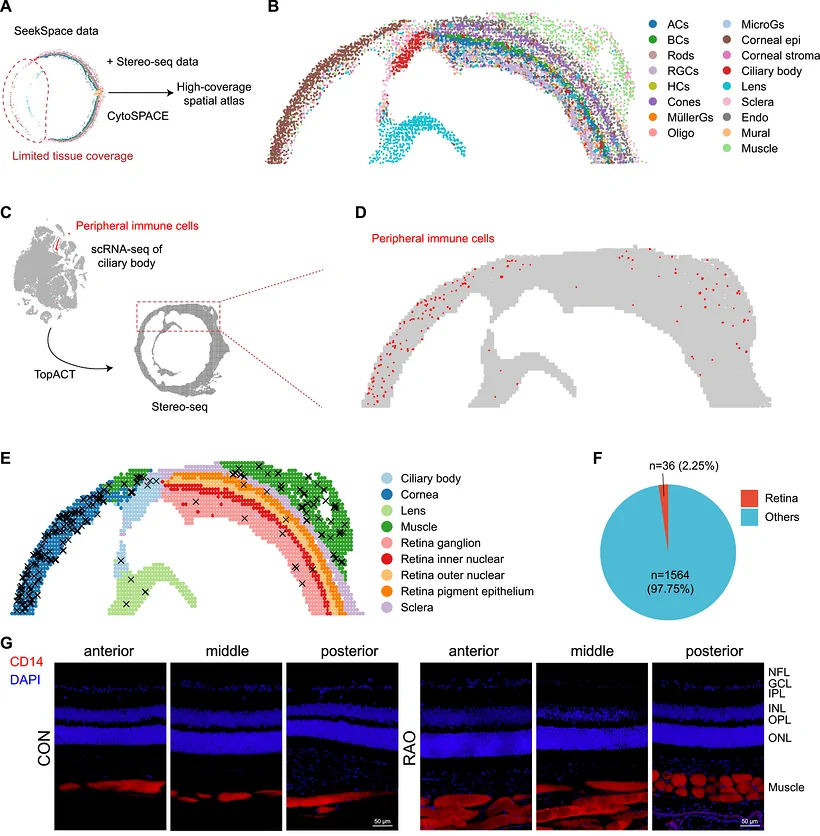
Reconstruction of single‐cell spatial localization based on Stereo‐seq. (A, B) Illustration (A) and predicted results (B) of the spatial localization for SeekSpace's major cell types using CytoSPACE based on Stereo‐seq at bin50 resolution. (C, D) Illustration (C) and predicted results (D) of the spatial localization for peripheral immune cells using TopACT based on Stereo‐seq at subcellular resolution, with red dots indicating predicted positions with a threshold of 0.7. (E) Spatial locations of TopACT‐predicted peripheral immune cells (black crosses, corresponding to the red dots in D) within Stereo‐seq derived clusters at bin50 resolution. (F) Pie chart showing the percentage of TopACT‐predicted peripheral immune cells that belong to the retina or not. (G) Representative images showing CD14 (red) and DAPI (blue) staining in the anterior, middle, and posterior segments of the eye from CON and RAO groups. The distinct layers of the retina are labeled as follows: nerve fiber layer (NFL), ganglion cell layer (GCL), inner plexiform layer (IPL), inner nuclear layer (INL), outer plexiform layer (OPL), and outer nuclear layer (ONL).

To address SeekSpace's low tissue coverage limitation, where sparse or absent cellular detection in certain regions compromised spatial comprehensiveness, we applied CytoSPACE [19] to align SeekSpace‐derived cell types onto Stereo‐seq sections to gain a comprehensive view (Figure 5A). For computational efficiency, we restricted subsequent analysis to one half of the eye, leveraging the organ's intrinsic bilateral symmetry. This integration generated a high‐coverage spatial atlas demonstrating precise anatomical correspondence of cell types (Figure 5B, Figure S5A).

Beyond tissue coverage, a second major limitation was the insufficient detection sensitivity for rare cell populations, which impeded accurate spatial mapping. To resolve this, we used Stereo‐seq as a comprehensive spatial reference with the TopACT [20] algorithm implementation. This enabled cell‐type‐specific localization of challenging cell types like diffusely distributed peripheral immune cells (Figure 5C). Analysis under both confidence thresholds (0.6 and 0.7) revealed sparse immune cell localization in similar specific regions (Figure 5D, Figure S5B). After aligning individual bin spot with bin50 annotations in a unified spatial coordinate, we unexpectedly observed a minimal infiltration of peripheral blood immune cells in retinal areas (Figure 5E, Figure S5C), with only 2.25% and 5.59% proportions at threshold 0.7 and 0.6, respectively (Figure 5F, Figure S5D). This computational mapping was further validated by immunofluorescence staining of CD14, which confirmed that peripheral immune cells are predominantly restricted to the extraocular muscle and surrounding connective tissues, with virtually no detectable signals within the retinal laminae (Figure 5G). This finding aligned with the blood‐retinal barrier's physiological properties [34], further confirming Stereo‐seq's reliability as a spatially resolved cellular annotation reference.

### 2.5 Müller Glia Identified by Automated Annotation but Not Unsupervised Clustering

**FIGURE 6:**
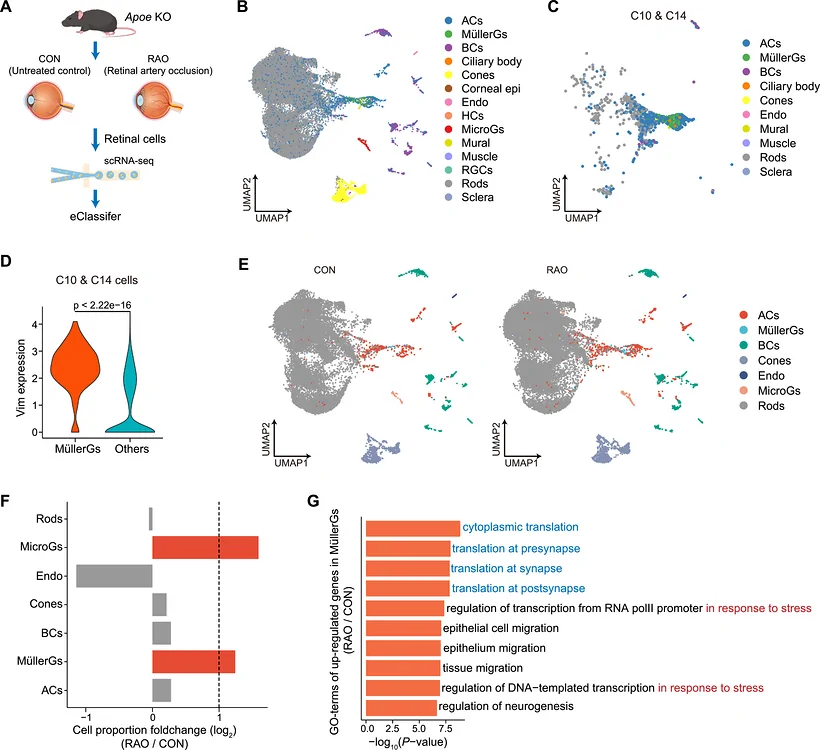
ASCAL pipeline enabling detection of the cell type missed by unsupervised clustering. (A) Illustration of experimental methodology for investigating the cellular and molecular changes of the retina upon RAO treatment. (B) UMAP projection of eClassifier‐predicted cell type annotation. (C) UMAP projection with a prominent display of C10 and C14 clusters with Müller glias, derived from eClassifier‐predicted cell type annotations. (D) Violin plot showing expression abundance of *Vim* between Müller glia and other cells in C10 and C14 clusters. The *P*‐values were calculated by the Wilcoxon test. (E) UMAP projection of annotated cell types in CON (control, left) and RAO (treatment, right) samples, respectively. (F) Bar plot showing cell proportion changes between RAO and CON. The cell types with over twofold changes are highlighted in red. (G) Bar plot showing the top 10 enriched GO‐terms (BP) of Müller glia up‐regulated genes in RAO.

RAO, a vision‐threatening ophthalmic emergency triggered by acute retinal artery obstruction‐induced ischemia and hypoxia, often leads to devastating visual impairment and requires urgent intervention. Thus, clarifying the eye's pathophysiological response to ischemia provides vital insights for developing sight‐saving therapeutics. Given the established association between hyperlipidemia and RAO [35, 36], we previously generated apolipoprotein E knockout (*Apoe* KO) mice to recapitulate this comorbidity. Here, we conducted scRNA‐seq on retinal tissues from *Apoe* KO mice subjected to UPOAO, a well‐established model of RAO [32], as well as from control eyes, to investigate molecular alterations induced by RAO under *Apoe*‐deficient conditions (Figure 6A). The *Apoe* KO dataset exhibited median values of 2,001 UMIs and 1,188 genes per cell (Figure S6A). Integration of *Apoe* KO and wild‐type (WT) mouse retinal datasets effectively removed technical batch effects (Figure S6B). Additionally, *Apoe* expression was markedly reduced in KO samples, confirming successful gene knockout (Figure S6C).

Application of the eClassifier (excluding the *Apoe* gene) to the KO dataset exhibited high concordance with unsupervised clustering, demonstrating its robustness in classifying genotype‐knockout samples (Figure 6B, Figure S6D,E). Notably, we identified Müller glial cells distributed across clusters 10 and 14 from unsupervised clustering (Figure S6E). Sub‐analysis of the two clusters demonstrated a concentrated distribution of Müller glial populations (Figure 6C), characterized by significantly higher expression of the canonical marker *Vim* compared to other cells in clusters 10 and 14 (Figure 6D, Figure S6F). The remaining clusters predominantly corresponded to distinct and unique cell types. Thus, we implemented a hybrid annotation approach, in which eClassifier‐annotated results in clusters 10 and 14 were retained, while other clusters were labeled based on dominant cell types, and cell types with cell number less than 50 were excluded (Figure 6E, Figure S6G,H). This highlighted the challenge of accurately resolving transcriptionally subtle or heterogeneous populations using unsupervised methods alone.

To rigorously evaluate the performance of eClassifier against unsupervised methods, we conducted a systematic parameter sweep across a range of clustering resolutions and neighborhood sizes. The results showed that the Jaccard index between the eClassifier‐annotated Müller glia and any unsupervised cluster remained exceptionally low, peaking at only 0.04 (max score of 1) across all 80 parameter combinations. This pattern was consistently observed in both the batch‐corrected integrated data and the standard normalized expression data, demonstrating the unique ability of eClassifier to robustly identify transcriptomically subtle cell populations that standard clustering pipelines might fail to detect (Figure S6I‐J).

Furthermore, extending this parameter sweep to other major retinal lineages confirmed that eClassifier aligns with intrinsic transcriptomic boundaries. Although unsupervised methods naturally fragmented highly heterogeneous populations such as ACs, BCs, and Rods into multiple subclusters, these subclusters consistently maintained high mean purities when mapped to eClassifier annotations (Figure S7). This systematic agreement demonstrates that eClassifier effectively overcomes the inherent limitations of unsupervised clustering by both rescuing ambiguous populations and seamlessly unifying overclustered lineages without relying on subjective parameter tuning.

To evaluate whether eClassifier's efficacy is independent of data resource, we conducted a direct comparison with SingleR [14], CellTypist [15], ScType [37], and TransferData [38] using identical datasets. UMAP visualizations revealed that eClassifier achieved superior global accuracy, whereas other methods exhibited notable assignment biases, such as CellTypist misclassifying major neuronal populations and ScType introducing non‐retinal noises (Figure S8A). Sankey diagrams further highlighted the pronounced classification instability of these alternative tools (Figure S8B). A critical commonality among SingleR, CellTypist, ScType, and TransferData was their inability to accurately isolate Müller glia. Quantitative evaluations confirmed that while these tools either missed this lineage entirely or generated excessive false positives, eClassifier successfully identified a biologically plausible population (Figure S8C). Finally, validation using the canonical marker *Vim* demonstrated that eClassifier maintained the highest marker enrichment and identification precision (Figure S8D). Collectively, these results demonstrate that the performance advantages of eClassifier stem from its superior method.

### 2.6 Immune Activation Induced by RAO Localized in GCL

Cellular proportion analysis revealed RAO‐induced expansions of microglia and Müller glia, consistent with immune activation (Figure 6F). Differential expression analysis identified the highest number of significantly altered genes in Müller glia (Figure S6K), with enriched GO‐terms related to translational regulation and stress response (Figure 6G). As the primary macroglial responders in the mammalian retina, Müller glia undergo pronounced reactive gliosis and functional remodeling under ischemic stress, playing an indispensable role in defining the spatial neuroinflammatory landscape alongside microglial activation.

Additionally, we performed Stereo‐seq on the eye sections from UPOAO model mice, enabling spatial transcriptomic analysis of disease‐associated molecular changes. To verify the UPOAO model, fundus photographs were captured using Micron IV Retinal Imaging Microscope during embolus insertion. Compared to the sham eye, the UPOAO fundus revealed narrowing of retinal vessels, whitening of arteries, cessation of venous blood flow, presence of a segmental flow void sign, and blood accumulation in the center of the pale optic disc (Figure S9A,B), confirming complete blockage of blood flow. The successful establishment of the UPOAO model was further validated by electroretinography (ERG) [39], which revealed a significant reduction in both a‐wave and b‐wave amplitudes in the UPOAO group compared to the Sham group, indicating substantial visual function impairment (Figure S9C,D). Moreover, this decline was more pronounced at 7 days compared to 3 days after reperfusion, suggesting progressive functional deterioration throughout the experiment.

**FIGURE 7:**
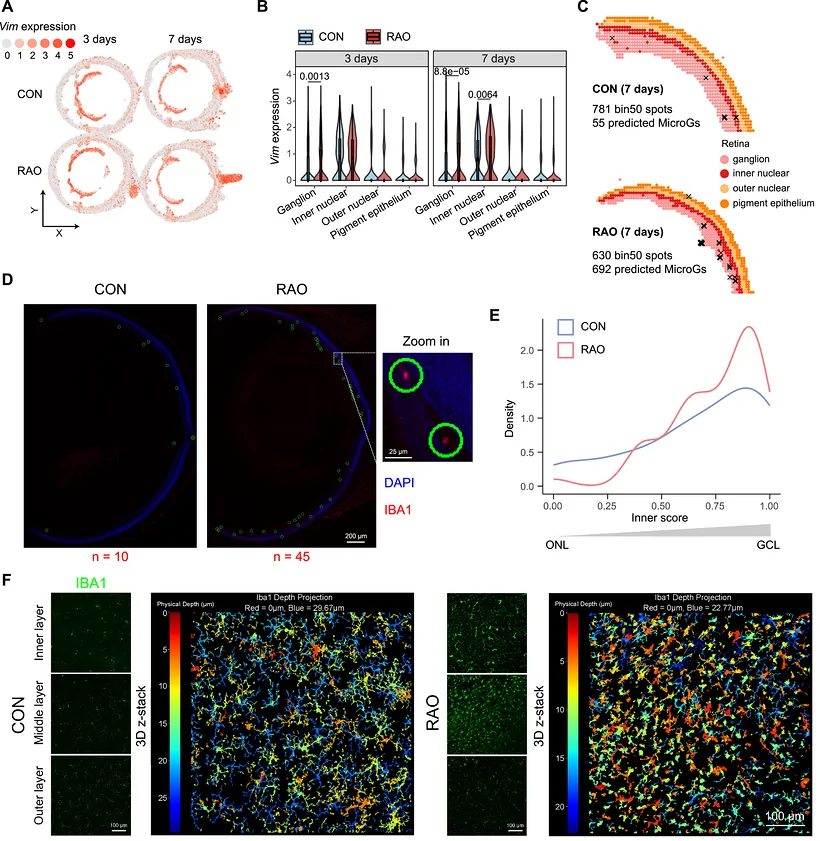
Spatial localization of immune activation induced by RAO. (A) Space coordinate plot showing *Vim* expression pattern in Stereo‐seq slices. (B) Violin plots quantifying *Vim* expression levels across distinct retinal anatomical layers in CON and RAO. In CON, the *P*‐values for comparing the ganglion cell and inner nuclear layers against the combined outer nuclear and pigment epithelium layers are 1.3e‐17 and 6.8e‐82, respectively. The marked *P*‐values represent the significance of differences between RAO and CON. The *P*‐values were calculated by the Wilcoxon test. (C) Spatial locations of TopACT‐predicted microglial cells (black crosses) within retina regions in CON and RAO slices (7 days) within Stereo‐seq derived clusters at bin50 resolution, respectively, with a threshold of 0.7. (D) Representative images showing IBA1 (red) and DAPI (blue) staining in the eye sections from CON and RAO groups. IBA1‐positive foci in the retina are demarcated by green circles. (E) Density plot showing the inner scores of IBA1‐positive foci in CON and RAO groups. (F) Representative images of IBA1‐labeled microglia in CON (left) and RAO (right) groups. Cross‐sectional views (top/middle/bottom panels) display microglial distribution across the inner, middle, and outer retinal layers, respectively. Global 3D z‐stack overlays on the right illustrate the overall microglial topography, with a pseudocolor bar indicating the spatial distance from the inner retina (red: inner; blue: outer).

Within the retinal tissue, the expression of the Müller glial cell marker gene *Vim* is strictly localized rather than representing a diffuse background signal. It is highly and specifically enriched in the ganglion cell layer (GCL) and the inner nuclear layer (INL) (Figure 7A,B). Notably, a quantitative comparison revealed significant *Vim* up‐regulation in the RAO model compared with controls. This increase initially appears in the GCL at 3 days post‐RAO. By day 7, the upregulation becomes significantly more pronounced and spreads across both the GCL and the INL, with the GCL showing the most notable increase (Figure 7B).

While Müller glial cells exhibited robust expression of the canonical marker *Vim* in Stereo‐seq sections, microglia‐specific marker signals were low at the spot level, making it difficult to detect microglia directly by examining marker gene expression (Figure S10A). To address this problem, we performed probabilistic cell‐type localization for single cells from snRNA‐seq based on Stereo‐seq, focusing the analysis on one half of the retinal region to improve computational efficiency. Strikingly, RAO‐treated samples at day 7 showed an over tenfold increase in predicted microglial hotspots compared to CON controls, despite having a smaller analyzed tissue area (as evidenced by fewer bin50 spots in the RAO section) (Figure 7C). This dramatic microglial expansion, predominantly localized to the GCL, corroborated the spatially restricted immune activation phenotype and highlighted the GCL as a pathological epicenter for neuroinflammatory responses following RAO injury.

To further validate the spatial distribution of immune activation, we performed immunofluorescence staining for the microglial marker IBA1 on ocular frozen sections and retinal flat‐mounts. Following RAO induction, a marked increase in IBA1‐positive signals was observed (Figure 7D). To quantify the spatial localization of these signals across the retinal laminae, we calculated an “inner score” for each immunoreactive focus, defined as the ratio of its distance to the outer boundary over the total retinal thickness (the sum of distances to both inner and outer boundaries). A higher inner score signifies a localization closer to the inner retina, representing the layers proximal to GCL. Statistical analysis revealed that the IBA1‐positive signals were significantly localized toward the inner retinal layers post‐RAO (Figure 7E), which is highly consistent with our computational findings.

In addition, we examined the distribution and morphology of microglia on retinal flat‐mounts. Consistent with the spatial distribution visualized in 3D z‐stack overlay and layer‐specific cross‐sectional images (Figure 7F), microglia in the control group were evenly distributed across the inner, middle, and outer retinal layers (Figure S11). In striking contrast, microglia in the RAO group displayed a marked redistribution toward the inner retinal layers (Figure S12). Concomitantly, microglia in the inner retina of RAO mice displayed shortened, thickened processes and reduced ramification, which are hallmarks of an activated phenotype, while those in the control group retained a highly branched, ramified morphology across all layers (Figure 7F). Notably, microglial density in the outer retinal layers was significantly diminished in RAO mice relative to controls (Figure 7F), further confirming the directional migration of microglia toward the inner retina following RAO insult.

Collectively, these data demonstrate that RAO triggers the migration of microglia toward the inner retina and their morphological transition to an activated state, characterized by process shortening and reduced ramification. This spatial redistribution and phenotypic shift suggest that inner retinal microglia may play a critical role in the early inflammatory response and pathological progression of retinal ischemia following RAO.

### 2.7 Topographic Distribution of Rod Subclusters

Building on this spatially restricted neuroinflammation, deconvolution analysis further demonstrated progressive neuronal loss, particularly RGCs, which decreased at 7 days post‐RAO. ERG showed a marked decrease in b‐wave amplitude in the UPOAO mouse model (Figure S9C,D), corresponding to the loss of BCs. Interestingly, Rods exhibited the smallest degree of change in overall abundance (Figure S13A). However, despite this overall stability in cell number, UMAP visualization suggested dynamic shifts in the distribution of Rods upon RAO treatment. Specifically, the RAO group exhibits a noticeable density shift and a marked reduction of a specific sub‐cluster at the bottom‐most region of the Rod cluster compared to the CON group (Figure 6E). This observation suggests a potential reorganization of Rod subcluster proportions following RAO.

**FIGURE 8:**
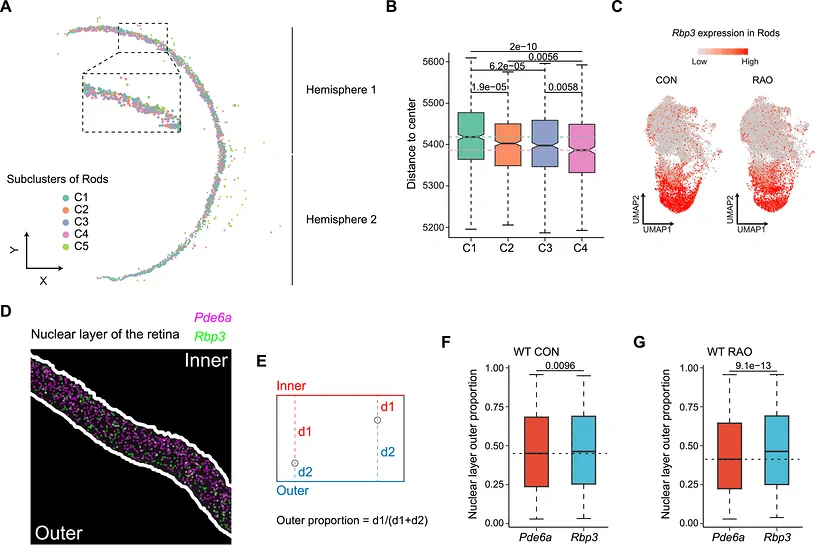
Spatial distribution of Rod subpopulations. (A) A tissue‐space coordinate plot showing subclusters of Rods within one SeekSpace section. The eye tissue is divided into two hemispheres. (**B**) Box plot showing the distribution of the distances between cells and the fitted eye center for each Rod subcluster. The *P*‐values were calculated by the Wilcoxon test. (C) UMAP projections showing the expression pattern of *Rbp3* in Rods under CON and RAO, respectively. (D) Representative field of the retinal nuclear layers visualized by RNAscope. Red circles indicate detected *Pde6a* signals, and green circles indicate detected *Rbp3* signals. (E) Illustration showing the calculation of the outer proportion for each detected signal. (F, G) Box plots showing the outer proportion of *Pde6a* and *Rbp3* signals in control (CON) (F) and RAO (G) eye sections from WT mice. The *P*‐values were calculated by the t‐test.

To directly interrogate spatial features of these subclusters, we leveraged SeekSpace's capacity for spatially resolved single‐cell mapping. Subcluster analysis identified five distinct Rod subgroups (Figure 8A), consistent with prior reports of Rod heterogeneity [40]. Among these, C5 displayed diffuse localization and was excluded from subsequent spatial analyses. The remaining subclusters, especially C1 and C4, exhibited distinct layered spatial patterns. To achieve quantitative characterization of spatial distribution across the subclusters, we located the eye center and calculated the radial distances from the center to individual cells. Statistical analysis revealed that C1 cells were generally located more peripherally, whereas C4 cells tended to cluster closer to the center (Figure 8B), consistent with the observed layering (Figure 8A). Furthermore, we observed asymmetric Rod subcluster distributions between the two hemispheres of the eye, with hemisphere 1 containing significantly more C4 cells than hemisphere 2, while hemisphere 2 has a higher proportion of C1 cells compared to hemisphere 1 (Figure S13B).

Given the relatively limited gene and cell detection throughput of SeekSpace, we extracted Rod cells from the scRNA‐seq dataset and classified them using eClassifier trained on Rod subclusters annotated in SeekSpace data (Figure S13C). We then calculated marker genes for the C1 subcluster relative to all Rods using the annotated scRNA‐seq dataset. Notably, *Rbp3* was specifically expressed in the C1 subcluster among Rods (Figure 8C). In SeekSpace tissue slices, the expression signal of *Rbp3* colocalized with that of the pan‐Rod marker *Pde6a*, but the *Rbp3* signal was distinctly enriched in the outer layer (Figure S13D), consistent with the spatial location of the C1 subcluster (Figure 8A,B). *Rbp3* encodes a key molecule involved in the transport of retinol and retinal within the visual cycle [41], and its high expression in C1 implies elevated visual cycle activity in this subpopulation.

To directly validate our computational findings, we performed RNAscope assays targeting the global Rod marker gene *Pde6a* and the C1 subcluster marker gene *Rbp3* to examine their spatial expression patterns. In the retinal nuclear layers, *Rbp3* signals were visibly positioned closer to the outer side compared with *Pde6a* (Figure 8D). Quantitative analysis, which measured the distance of each signal to the inner and outer boundaries of the layer and calculated the outer proportion (Figure 8E), confirmed that *Rbp3* signals were consistently located more peripherally than *Pde6a* (Figure 8F,G). This positional shift was observed in both WT and *Apoe* KO mice under control conditions as well as in RAO‐induced sections (Figure S13E).

### 2.8 A State‐Dependent Vulnerability Within Rods Induced by RAO

**FIGURE 9:**
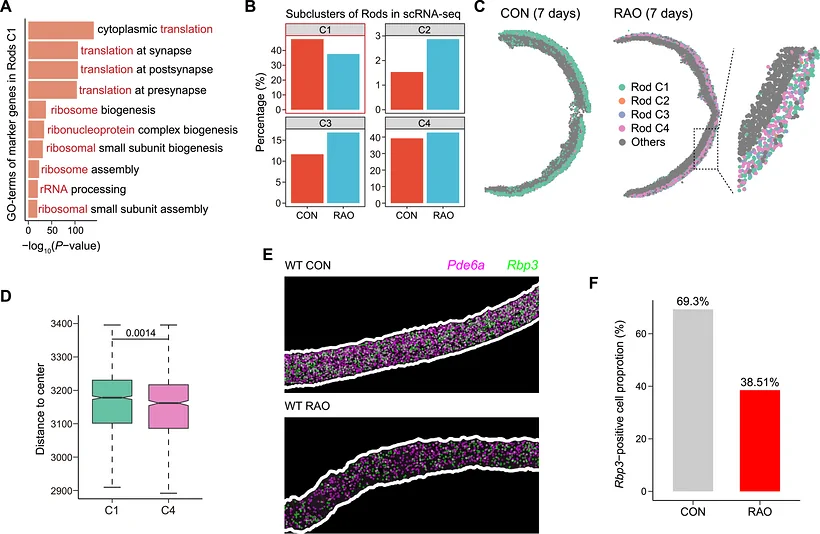
Alterations of Rod subpopulations associated with RAO treatment. (A) Bar plot showing the top 10 enriched GO‐terms (BP) of marker genes in C1 subcluster among Rods. (B) Bar plots showing the relative proportions of 4 Rod subclusters among total Rods in CON and RAO. (C) Predicted spatial localization of Rod subclusters in retina using CytoSPACE based on Stereo‐seq at bin50 resolution in CON (left panel) and RAO (right panel), respectively. (D) Box plot showing the distribution of the distances between cells and the fitted eye center for Rod C1 and C4 subclusters. The *P*‐values were calculated by the Wilcoxon test. (E) Representative fields of the retinal nuclear layers visualized by RNAscope. Red circles indicate detected *Pde6a* signals, and green circles indicate detected *Rbp3* signals. (F) Bar plot showing the proportion of *Rbp3*‐positive cells in control (CON) and RAO eye sections of WT mice.

In addition to *Rbp3*, the C1 marker genes were significantly enriched for translation‐related biological processes (Figure 9A), including multiple ribosomal protein genes such as *Rps15a* (Figure S13F). This enrichment suggested that the C1 subcluster was highly active in protein synthesis, likely underpinning its specific functional state. Next, to determine whether Rod subpopulation composition was altered despite a stable total abundance, eClassifier‐based scRNA‐seq annotation revealed a marked reduction in the proportion of the C1 Rod subcluster following RAO (Figure 9B). When mapping single‐cell data to Stereo‐seq sections using CytoSPACE, with CON and RAO single cells mapped to their respective day 7 sections, we consistently observed marked differences in the spatial composition of Rod subclusters. In the control section, the Rod C1 subpopulation was predominant, whereas in the RAO section, there was a substantial emergence of the C4 subpopulation (Figure 9C). Notably, within RAO sections, the C1 subcluster was more peripherally localized (Figure 9D), in line with the aforementioned findings. Moreover, analysis of previously published bulk RNA‐seq data from RAO‐treated mouse retinas [32] revealed significant down‐regulation of several C1 Rod subcluster marker genes at 7 days post‐RAO (Figure S13G), including several ribosomal genes, such as *Rps15a* (Figure S13F), suggesting an association with the loss of this subpopulation after RAO treatment.

In the RNAscope validation, we also observed a clear reduction in the number of *Rbp3* signals in RAO samples compared with controls of WT mice (Figure 9E). Quantitative analysis, in which *Rbp3* signal counts were normalized to the number of nuclei calculated from the DAPI signals, showed that this proportion was reduced by nearly half in WT mice after RAO (Figure 9F). These findings further corroborated the computational conclusion that the active Rod subpopulation was selectively depleted following RAO.

Collectively, our ASCAL‐based analysis demonstrates that RAO triggers retinal immune activation predominantly localized to the GCL, while maintaining total Rod abundance but selectively reducing a functionally and translationally active Rod subcluster located in the outer layer. These results provide new cellular and molecular insights for mechanistic studies of RAO and related diseases.

## 3 Discussion

RAO induces a spatially heterogenous cascade of retinal injury, characterized by region‐specific immune activation, selective neuronal loss, and glial responses, processes that are challenging to dissect with conventional sequencing methods. In this study, we developed the ASCAL computational pipeline to integrate spatial information from two complementary modalities: single‐cell resolution SeekSpace data for precise expression reference and subcellular‐resolution Stereo‐seq data for comprehensive spatial reference (Figure 1C), both of which operate independently of cell segmentation. This strategy takes advantage of SeekSpace's single‐cell resolution and Stereo‐seq's ability to detect uniform signals at subcellular resolution. For Stereo‐seq, ASCAL employs a multi‐scale strategy where a binning approach (bin50) is utilized to characterize the global ocular architecture. This resolution is sufficient to capture the organizational complexity of the eye, while the aggregation enhances gene‐capture efficiency and sensitivity in identifying robust tissue‐specific signatures. Simultaneously, binning constraints are bypassed for rare cell populations by applying the TopACT algorithm to prevent the dilution of sparse signals, thereby maintaining high‐precision localization at the subcellular level. By combining these strengths, ASCAL enables high‐accuracy, automated cell‐type annotation and spatial mapping of scRNA‐seq datasets. Notably, the approach utilizes only a limited amount of spatial transcriptomics data (tissue slices) as a high‐confidence reference to annotate and precisely localize large‐scale, high‐throughput scRNA‐seq datasets, thereby avoiding the need for exhaustive spatial transcriptomics. It is particularly advantageous in regions with overlapping cell morphologies or densely packed cells, where segmentation‐based methods often encounter assignment errors. Furthermore, the integrative strategy offers a scalable alternative to labor‐intensive laser capture microdissection (LCM) methods [42, 43], enabling high‐throughput, whole‐tissue spatial expression reference construction for understudied tissues. Critically, this approach eliminates the requirement for prior knowledge‐dependent iterative LCM harvesting, which necessitates manual selection and isolation of individual regions, instead achieving systematic and unbiased tissue characterization via a single SeekSpace assay.

The ASCAL workflow effectively handles modality‐specific effects and tissue heterogeneity, which is essential for integrating SeekSpace, Stereo‐seq, and single‐cell RNA‐seq data. At the computational level, we mitigated systemic biases by retaining only co‐expressed genes during classifier training and employing a mapping strategy based on global correlation and probabilistic models. This approach avoids direct comparisons of absolute transcript counts, thereby overcoming inherent differences in capture efficiency among different technologies. At the spatial level, we addressed tissue deformation by using H&E‐guided selection of intact sections and establishing the Stereo‐seq slices as a global spatial scaffold. By training eClassifer with SeekSpace data, annotating individual cells from scRNA‐seq, and computationally anchoring those cells to this scaffold, instead of attempting rigid physical alignment, we constructed a reliable spatial reference system that maintains macroscopic coherence while achieving single‐cell precision.

To demonstrate the practical utility of ASCAL, we selected the mouse eye as a representative application scenario, performing spatial transcriptomics on 7 ocular sections and scRNA‐seq on dissociated tissues (Figure 1A). The eye is a structurally complex and spatially heterogeneous organ, which presents significant challenges for the construction of spatial transcriptomic atlases at the single‐cell level. First, we utilized SeekSpace technology to establish a single‐cell resolution spatial atlas of the mouse eye, effectively overcoming technical limitations imposed by the high heterogeneity of ocular tissues. By leveraging SeekSpace data, we simultaneously captured both expression profile and spatial positioning for single cells, enabling precise annotation of cell types. Subsequently, we employed SeekSpace data as a reference to construct an SVM classifier (eClassifier) for automated annotation of single‐cell datasets. This approach eliminated biases associated with manual marker gene expression assessment and addressed the challenge of annotating non‐retinal regions lacking well‐defined marker genes.

To validate the accuracy of eClassifier in ocular cell classification, we performed cross‐validation against unsupervised clustering results, achieving over 85% concordance in cell‐type assignments. Furthermore, tissue‐specific cell populations were accurately identified through eClassifier in scRNA‐seq datasets from distinct tissues. The eClassifier enabled precise mapping of rare cell subtypes, which were overlooked by traditional unsupervised clustering methods, demonstrating the robustness of eClassifier in resolving both broad anatomical regions and functionally distinct cellular niches within the eye. To further refine spatial localization, we incorporated Stereo‐seq, a subcellular resolution spatial transcriptomic platform, to achieve precise spatial mapping of identified cell types. The accuracy of the spatial map was validated by the correct localization of cell types to their expected positions.

Although eClassifier demonstrates significant advantages in cell type annotation for single‐cell transcriptomics, several limitations should be acknowledged. Our current evaluation primarily utilizes cluster purity as the core metric. While high purity scores validate the algorithm's robustness in capturing established biological lineages, this metric primarily measures classification consistency rather than annotation accuracy, which requires a ground truth. A comprehensive evaluation of eClassifier is merited across a variety of datasets from diverse tissues, assessing annotation accuracy, computational speed, memory usage, etc.

Additionally, utilizing the ASCAL pipeline, we spatially uncovered RAO‐induced molecular alterations at single‐cell resolution in the mouse retina, including pronounced immune activation and dynamic shifts in Rod substate composition. Specifically, this included a predominant immune response localized to the retinal ganglion cell layer, and a reduction of a functionally active Rod subcluster situated in the outer Rod layer under RAO treatment, despite stable total Rod abundance. This selective depletion was further validated by RNAscope assays, which confirmed both the peripheral localization of this subcluster and its marked loss after RAO. While bulk tissue and single‐cell transcriptomic analyses in the murine retinal ischemic‐reperfusion model revealed global molecular alterations [32], these approaches lack spatial resolution to interrogate region‐specific pathologies, a critical limitation given the localized progression of retinal degenerative processes. To address this gap, our study utilized ASCAL to integrate spatial transcriptomics, uncovering compartmentalized pathology that was not detectable by previous methods. These findings significantly advance the mechanistic understanding of disease progression and lay the groundwork for future therapeutic targeting of localized degenerative cascades.

Our study presents the first spatially resolved whole‐eye atlas at single‐cell resolution in mice, achieved through the ASCAL integration strategy. This resource comprehensively mapped molecular features across all ocular substructures, including the neural retina and auxiliary tissues, thus bridging historical investigations of individual eye components. Two recent studies employed Visium Spatial Gene Expression (10x Genomics) to construct a valuable spatial transcriptomic atlas of the mouse eye [44], and to map human retinal development [45], respectively. While these foundational works provide important insights into ocular organization, the platform‐inherent resolution constraints limit detailed characterization of retinal substructures. Some studies have also applied the high‐resolution MERFISH technology to construct single‐cell spatial atlases of the mouse retina [46]. Nevertheless, the extreme cellular and organizational heterogeneity of ocular tissues, particularly within the retina, resulted in distinct anatomical region‐specific cell compositions. These variations posed significant challenges for image segmentation algorithms in accurately delineating cell boundaries. SeekSpace overcame these limitations by achieving physical single‐cell resolution through the sequential addition of spatial and cell barcodes. This method eliminated reliance on error‐prone computational cell segmentation and ensured precise single‐cell isolation without RNA cross‐contamination.

Despite achieving single‐cell spatial resolution, constructing a single‐cell reference atlas exclusively from SeekSpace data remains constrained by inherent limitations. First, rare or sparsely distributed cell types, such as peripheral immune cells, may not be adequately captured due to limited cell counts within thin tissue sections. These populations are typically better represented in dissociated tissue‐derived scRNA‐seq datasets, necessitating manual curation before the ASCAL workflow. Furthermore, spatial signal distribution in SeekSpace exhibits non‐uniformity. Since this platform relies on the physical isolation of intact nuclei, tissue sectioning may disrupt nuclear integrity, resulting in reduced capture efficiency, especially for cells with larger nuclei. Conversely, Stereo‐seq employs the RNA permeation‐based transcript capture method, circumventing the need for intact nuclei and thereby achieving broader and more uniform transcript coverage. This complementary strength enables Stereo‐seq to mitigate SeekSpace's limitations in achieving uniform spatial coverage and provides a more complete spatial reference for comprehensive cell‐type positioning.

In sum, the ASCAL approach holds broad applicability for spatial transcriptomic studies in poorly characterized tissues, particularly those with poorly annotated cell types or ambiguous marker genes. By integrating multi‐dimensional data, our strategy advanced the study of complex anatomical systems with reduced reliance on large‐scale spatial transcriptomics and paved the way for systematic exploration of spatially regulated biological processes in disease and regeneration. Notably, in the context of RAO, ASCAL uncovered critical insights such as spatially restricted immune activation in the ganglion cell layer and selective depletion of a functionally active Rod subcluster, which enhance our understanding of RAO's compartmentalized pathology. Future adaptations could extend to clinical samples or cross‐species analyses, further democratizing spatially resolved omics in resource‐limited settings. We recommend ASCAL for tissues that lack well‐defined molecular markers but possess clear anatomical structures, as it enables accurate annotation and spatial localization in single‐cell analyses, thereby improving reliability even without large‐scale spatial datasets.

## 4 Methods

### 4.1 Animal Studies

Wild‐type C57BL/6J and *Apoe* knockout (*Apoe* KO) mice of male sex (aged 8 weeks) were used for the experiments. Only male mice were used to exclude the potential confounding effects of estrogen. All mice had sufficient food and water at a 12‐hour dark‐light cycle in a room with regulated temperature conditions. All animal procedures were performed in accordance with the ethical guidelines of the Association for Research in Vision and Ophthalmology (ARVO) Statement for the Use of Animals in Ophthalmic and Vision Research and were approved by the Experimental Animal Ethics Committee of Renmin Hospital of Wuhan University (approval number: WDRM‐202500130). Mice used in this study included C57BL/6J (RRID: IMSR_JAX:000664) and Apoe‐deficient mice (RRID: IMSR_JAX:002052), which were obtained from Liaoning Changsheng Biotechnology Co., LTD.

### 4.2 Unilateral Pterygopalatine Ophthalmic Artery Occlusion Model

In this study, the RAO model was established via unilateral pterygopalatine ophthalmic artery occlusion (UPOAO), a well‐established experimental approach to mimic retinal ischemia. UPOAO was performed as previously described [32]. Briefly, mice were anaesthetized with a 2% concentration of isoflurane delivered in a mixture of nitrous oxide and oxygen. Body temperature was maintained at 37 ± 0.5°C throughout the surgery by using a heating blanket. A midline neck incision was made. After separating the neck gland, the left common carotid artery (CCA), external carotid artery (ECA), and internal carotid artery (ICA) were isolated and ligated with a 6‐0 suture. A slipknot was created at the proximal end of ECA. A silicone wire embolus measuring 7 ± 0.1 mm in length and 0.21 ± 0.1 mm in diameter was inserted into the CCA via an incision between the two suture knots on the ECA. The slipknot on ECA was then tightened, and the ligation on the ICA was removed. Subsequently, the embolus advanced counterclockwise through the ICA and further into the pterygopalatine artery (PPA), effectively obstructing the ophthalmic artery. The PPA was occluded for 1 h. After occlusion, the animals were re‐anesthetized, and the embolus was carefully removed. Finally, the CCA ligation was removed, restoring arterial reperfusion. After recovery, the mice were kept in their home cage with facilitated access to water and food.

### 4.3 Electroretinogram

After reperfusion for 3 days and 7 days, the Electroretinogram (ERG) measurements were performed to determine whether the UPOAO model was successful. Mice underwent overnight dark adaptation, and all subsequent procedures were conducted under dim red light or complete darkness. The mice were anaesthetized via intraperitoneal injection of 1% sodium pentobarbital and then subjected to pupil dilation using tropicamide for 5 min. Then, a subcutaneous electrode was inserted into the posterior cervical skin, a tail electrode was affixed to the tail, and the corneal electrodes were gently positioned on the center of both corneas. Electrical responses were recorded using a Retina‐C Visual Electrophysiology System (Shanghai 3VMED Co., Ltd.). Dark‐adapted a‐waves, b‐waves, and oscillatory potentials (OPs) were recorded under stimulus intensities of 0.01, 0.03, 0.1, 0.3, 1, 3, and 10 cd·s/m^2^. The latencies and amplitudes of a‐waves, b‐waves, and OPs in response to various flash stimuli were analyzed for each subject.

### 4.4 Sample Collection for SeekSpace and Stereo‐seq

After reperfusion for 3 days and 7 days, fresh mouse eyeball samples were obtained within 5 min of cervical dislocation, embedded in one container filled with optimum cutting temperature (OCT) compound (SAKURA, USA), and frozen at −80°C. Frozen sectioning was performed at −20°C in the direction parallel to the optic nerve by using a freezing microtome (Leica, Wetzlar, Germany). When the tissue was exposed, the sections were collected for RNA extraction and quality check. After trimming the frozen tissue block by approximately 1500 µm, one 4‐µm‐thick section was obtained for HE staining, and one 10‐µm‐thick frozen section was adhered to a chip surface for further experiments.

### 4.5 Cell Nuclei With Spatial Barcodes Preparation for SeekSpace

Single‐cell nuclei suspension with spatial barcodes was prepared from fresh frozen tissues using the SeekSpace Single Cell Spatial Transcriptome‐seq Kit (K02501‐08). Briefly, fresh frozen tissues were cryo‐sectioned to 10 µm on a cryostat (Leica) at −20°C. The tissue regions of interest were then placed on the SeekSpace Chip, ensuring there were no folds. A finger was placed on the back of the SeekSpace Chip to melt the tissue. The SeekSpace Chip was then placed in the SeekSpace sc‐Spatial Chip Holder and incubated on a Thermocycler Adaptor at 37°C for 90 s. Then, a Space Chamber was placed on the chip, and 150 µl of labeling reagent was added without introducing bubbles. Next, the tissue sections were fixed, fluorescence photographed, and homogenized in pre‐chilled lysis buffer using a Dounce homogenizer (KIMBLE #D8938). After washing and filtration, the number of nuclei was estimated using a Fluorescence Cell Analyzer (Seekgene#M002B) with AO/PI reagent, and then the sample was placed on ice for further use.

### 4.6 Sequencing Library Preparation for SeekSpace

Single‐cell RNA‐Seq libraries and spatial barcode libraries were prepared using the SeekSpace Single Cell Spatial Transcriptome‐seq Kit (K02501‐08) according to the manufacturer's instructions. Briefly, the nuclei were evenly divided into 8 PCR tubes, and reverse transcription was carried out on 600–30 000 nuclei in each PCR tube, with a different labeled reverse transcription primer added to each tube. Fifteen cycles of annealing (ramping from 8°C to 42°C) were performed to enhance primer hybridization and intracellular reverse transcription efficiency. After reverse transcription, the nuclei were washed twice to remove residual primers and pooled together. Subsequently, an appropriate number of nuclei were combined with ligation reagents and added to the sample wells of the SeekOne DD Chip S3 (Chip S3). Barcoded Hydrogel Beads (BHBs) and partitioning oil were then dispensed into the corresponding wells separately in Chip S3. The cell‐containing ligation reagents and BHBs were encapsulated into emulsion droplets using the SeekOne Digital Droplet System. Immediately after transferring the emulsion droplets into PCR tubes, a 60‐minute incubation at 20°C followed by a 10‐min heat inactivation at 65°C were performed to obtain barcoded cDNA and spatial barcodes. The barcoded cDNA and spatial barcodes were then decrosslinked and recovered from cells in droplets. To obtain more Template‐Switched cDNA, a second reverse transcription was performed, followed by a PCR pre‐amplification. The pre‐amplified product was used as input for both spatial barcode library construction and cDNA construction. Finally, sample indexes were added to the pre‐amplified product during spatial barcode library construction via PCR. After cDNA purification, 20 ng of cDNA was amplified by index PCR. The indexed sequencing libraries were cleaned with VAHTS DNA Clean Beads (Vazyme N411‐01), analyzed by Qubit (Thermo Fisher Scientific Q33226) and Bio‐Fragment Analyzer (Bioptic, Qsep400). The single‐cell RNA‐Seq libraries and spatial barcode libraries were then sequenced on the Illumina NovaSeq X Plus with a PE150 read length.

### 4.7 Library Construction and Sequencing of Stereo‐seq

Tissue sections were adhered to the Stereo‐seq chip (generated by BGI, China) surface and incubated at 37°C for 3 min. Then, the sections were fixed in methanol and incubated for 40 min at −20°C before Stereo‐seq library preparation. Where indicated, the same sections were stained with nucleic acid dye (Thermo fisher, Q10212) and imaging was performed with a Motic Custom PA53 FS6 microscope prior to in situ capture at the channel of FITC. After washed with 0.1x SSC buffer (Thermo, AM9770) supplemented with 0.05 U/ml RNase inhibitor (NEB, M0314L), tissue sections placed on the chip were permeabilized using 0.1% pepsin (Sigma, P7000) in 0.01 m HCl buffer, incubated at 37°C for 5 min and then washed with 0.1x SSC buffer (Thermo, AM9770) supplemented with 0.05 U/mL RNase inhibitor (NEB, M0314L). RNA released from the permeabilized tissue and captured by the DNB was reverse transcribed overnight at 42°C using SuperScript II (Invitrogen, 18064‐014, 10 U/mL reverse transcriptase, 1 mm dNTPs, 1 M betaine solution PCR reagent, 7.5 mm MgCl_2_, 5 mm DTT, 2 U/ml RNase inhibitor, 2.5 mm Stereo‐seq‐TSO, and 1x First‐Strand buffer). After reverse transcription, tissue sections were washed twice with 0.1x SSC buffer and digested with Tissue Removal buffer (10 mm Tris‐HCl, 25 mm EDTA, 100 mm NaCl, 0.5% SDS) at 55°C for 10 min. cDNA‐containing chips were then subjected to Prepare cDNA Release Mix (cDNA Release Enzyme, cDNA Release buffer) treatment for overnight at 55°C. cDNA was purified using the VAHTSTM DNA Clean Beads (0.8x).

The resulting cDNAs were amplified with KAPA HiFi Hotstart Ready Mix (Roche, KK2602) with 0.8 mm cDNA‐PCR primer. PCR reactions were conducted as follows: incubation at 95°C for 5 min, 15 cycles at 98°C for 20 s, 58°C for 20 s, and 72°C for 3 min, followed by a final incubation at 72°C for 5 min.

The concentrations of the resulting PCR products were quantified by QubitTM dsDNA Assay Kit (Thermo, Q32854). A total of 20 ng of DNA was then fragmented with in‐house Tn5 transposase at 55°C for 10 min, after which the reactions were stopped by the addition of 0.02% SDS and gently mixing at 37°C for 5 min after fragmentation. Fragmented products were amplified as described below: 25 mL of fragmentation product, 1x KAPA HiFi Hotstart Ready Mix, and 0.3 mm Stereo‐seq‐Library‐F primer, 0.3 mm Stereo‐seq‐Library‐R primer in a total volume of 100 mL with the addition of nuclease‐free H_2_O. The reaction was then run as: 1 cycle of 95°C 5 min, 13 cycles of 98°C 20 s, 58°C 20 s, 72°C 30 s, and 1 cycle of 72°C 5 min. PCR products were purified using the AMPure XP Beads (0.63 and 0.153), used for DNB generation, and finally sequenced on the MGI DNBSEQ‐Tx sequencer.

### 4.8 Retinal Sections Immunofluorescence

Mice eyeballs were enucleated after cervical dislocation. The eyeballs were then embedded in OCT compound and sectioned into 14‐µm‐thick slices parallel to the optic nerve. Serial sections were collected at the following three positions: 1) 1/3 of the eyeball length: 6 sections were collected starting from 960 µm, labeled as “anterior”; 2) 1/2 of the eyeball length or at the optic disc: 6 sections were collected starting from 1430 µm, labeled as “middle”; 3) 2/3 of the eyeball length: 6 sections were collected starting from 1960 µm, labeled as “posterior”.

Immunofluorescence procedure was performed as follows: After fixation of the frozen sections, antigen retrieval was carried out using Tris‐EDTA retrieval buffer (pH 8.0, Servicebio, Cat. No. G1206) in an antigen retrieval instrument (Servicebio, Model ARI‐4) for 4 h. The sections were then blocked with PBS containing 5% bovine serum albumin (BSA) and 0.5% Triton X‐100 at room temperature for 1 h. Subsequently, the sections were incubated overnight at 4°C with primary antibodies against CD14 (1:1000 dilution, abcam, Cat. No. EPR21847) or IBA1 (1:500 dilution, Servicebio, Cat. No. GB113502). After three washes with PBS, the sections were incubated with CY3‐conjugated goat anti‐rabbit IgG secondary antibody (1:300 dilution, Servicebio, Cat. No. GB21303) at room temperature in the dark for 1 h. Following another three washes with PBS, the sections were stained with DAPI solution at room temperature in the dark for 10 min. After gently shaking off excess liquid, the sections were treated with autofluorescence quencher (Servicebio, Cat. No. G1221) for 5 min, rinsed with running water for 10 min, and then mounted with an anti‐fade mounting medium (Servicebio, Cat. No. G1401) and coverslipped. Images were acquired using a digital tissue section scanner (3DHISTECH, Model Pannoramic MIDI) with the following excitation and emission wavelengths: DAPI (excitation wavelength: 330–380 nm, emission wavelength: 420 nm) and CY3 (excitation wavelength: 510–560 nm, emission wavelength: 590 nm).

IBA1‐positive signals were identified using a Difference of Gaussians (DoG) algorithm. Briefly, the original images were processed with two Gaussian kernels (sigma = 2.0 and sigma = 4.0) to define the foreground signal and background noise, respectively. The resulting DoG map was thresholded at a value of 5/255 (normalized intensity). To ensure the biological specificity of the detected immunofluorescent foci, a size filter was subsequently applied to exclude excessively small, non‐specific background artifacts.

### 4.9 Retinal Flat‐Mounts Immunofluorescence of Microglia

Mice were sacrificed by cervical dislocation, and the eyeballs were immediately enucleated and fixed in 4% paraformaldehyde (PFA) at room temperature for 60 min. After fixation, the cornea and lens were carefully excised, and the intact retina was dissected. The isolated retinas were subjected to permeabilization and blocking by incubation in PBS containing 5% BSA and 0.5% Triton X‐100 at 4°C overnight, followed by incubation with the primary antibody against IBA1 (1:500, GeneTex, Cat. No. GTX635363) at 4°C for 2 days. After primary antibody incubation, the retinas were gently rinsed three times with PBS for 20 min each to remove unbound antibody. The retinas were then incubated with Alexa Fluor 488‐conjugated donkey anti‐rabbit secondary antibody (1:500, AntGene, Cat. No. ANT025) at 4°C for 1 day in a light‐protected container to prevent fluorophore quenching. Following another three rinses with PBS, the retinas were evenly cut into a four‐leaf clover shape. Subsequently, the retinas were mounted with anti‐fluorescence quenching mounting medium containing DAPI and flattened with coverslips. Z‐stack scanning of the Iba1‐labeled retinal flat‐mounts was performed using a confocal laser scanning microscope (Leica TCS SP8, Germany), and 3D reconstruction of the microglial morphology was further conducted based on the acquired z‐stack images.

### 4.10 SeekSpace Data Analysis

The SeekSpace Tools (v1.0.0) were used to conduct data quality analyses of the SeekSpace sequencing data. Reads were mapped to the mouse mm10 genome assembly using STAR [47]. The UMI count matrix was processed using the R package Seurat using the CreateDimReducObject function to add a spatial coordinate for each cell. The object was sequentially normalized, scaled, subjected to dimensional reduction, clustered, and annotated in the Seurat package. The FindAllMarkers function was then used to identify cell‐type‐specific marker genes.

### 4.11 Stereo‐seq Data Analysis

Fastq files were generated using the MGI DNBSEQ‐Tx sequencer and were processed through the SAW pipeline (https://github.com/STOmics/SAW). CID and MID are contained in read 1 (CID: 1–25 bp, MID: 26–35 bp) while read 2 consists of the cDNA sequences. CID sequences on the first reads were first mapped to the designed coordinates of the in situ captured chip achieved from the first round of sequencing, allowing for a 1‐base mismatch to correct for sequencing and PCR errors. Reads with MID containing either N bases or more than 2 bases with the quality score lower than 10 were filtered out. CID and MID associated with each read were appended to each read header. Retained reads were then aligned to the mm10 reference genome using STAR [47], and mapped reads with MAPQ > 10 were counted and annotated to their corresponding genes. UMIs with the same CID and the same gene locus were collapsed, allowing for a 1‐base mismatch to correct for sequencing and PCR errors. Finally, this information was used to generate a CID‐containing expression profile matrix.

Bin50 data was read in through the Seurat (v.4.3.0) package in R, and normalized using the NormalizeData function. The FindVariableFeatures function was then applied to identify 2000 highly variable features, which were subsequently used for principal component analysis (PCA). The top 10 principal components were selected to construct the KNN network via the FindNeighbors function, followed by clustering using the FindClusters function at a resolution of 0.35. The resulting clusters were manually annotated based on anatomical position. Cluster‐specific genes were identified using the FindAllMarkers function, applying a foldchange threshold greater than 2 and a *P*‐value cutoff of less than 0.05.

### 4.12 Identification of Tissue‐Specific Genes

To distinguish high‐confidence tissue‐specific genes from general differentially expressed genes, a stringent set of selection criteria was implemented using the Seurat (v.4.3.0) FindAllMarkers function. Genes were defined as tissue‐specific only if they concurrently satisfied an adjusted *P*‐value < 0.05, a log2 fold change > 2, and a maximum detection rate of less than 5% (pct.2< 0.05) in all other clusters. This strict pct.2 constraint ensured the identified markers were uniquely or predominantly restricted to the target tissue compartment.

### 4.13 Single‐Cell RNA‐seq Data Analysis

For single‐cell data integration, raw gene expression matrices from multiple samples were first read in and merged into a Seurat object. Single‐cell quality control was performed by excluding cells with extreme outliers in gene and UMI counts. Individual datasets were independently normalized using the “SCTransform” algorithm. Integration was performed via the Seurat pipeline by identifying anchors across datasets, followed by reciprocal projection to harmonize batch effects. The integrated data were scaled, and principal component analysis (PCA) was applied for dimensionality reduction, enabling downstream unsupervised clustering and UMAP or tSNE visualization. Next, we applied the “FindMarkers” function to perform differential analysis on RAO and CON samples. Genes exhibiting a foldchange greater than 1.5 and *P*‐value less than 0.05 were identified as differentially expressed genes (DEGs).

### 4.14 Spatial Neighborhood Enrichment Analysis

To quantify cluster‐space correspondence, we constructed a spatial neighbor graph by identifying the 10 nearest physical neighbors for each cell based on their in situ coordinates. A null distribution was generated by randomly shuffling cluster labels 100 times. For each cell‐type pair, an enrichment Z‐score was then calculated by comparing the observed frequency of spatial adjacency against the expected frequency from the null distribution, providing a localized assessment of spatial clustering.

### 4.15 Construction of an SVM Classifier for Eye Cells (eClassifier)

Support Vector Machine (SVM) analysis was performed using the svm function from the e1071 R package (v.1.7‐14). The SVM classifier was trained using scaled (normalized and variance‐stabilized) data from all cells in the SeekSpace reference data, with cell‐type labels derived from annotations. For cell‐type prediction, query single‐cell datasets were preprocessed identically, and only genes co‐expressed in both the SeekSpace reference and query data were retained for model input (*Apoe* gene was excluded in the *Apoe*‐knockout dataset). The trained SVM model was then applied to annotate cell types of query datasets based on the scaled data.

### 4.16 Quantitative Evaluation of Clustering Robustness

To evaluate the capture efficiency of Müller glia across different clustering strategies, we performed a systematic parameter sweep using unsupervised Seurat clustering. We tested a grid of 80 parameter combinations, including 20 resolution settings ranging from 0.1 to 2.0 and 4 neighborhood sizes (k = 10, 20, 30, and 50). For each combination, the Jaccard index was calculated to quantify the overlap between the eClassifier‐annotated Müller glia and the single unsupervised cluster that showed the highest degree of similarity. This analysis was performed on both the batch‐corrected integrated data and the standard normalized expression data to ensure the consistency of our findings.

Furthermore, to assess the clustering performance for highly heterogeneous cell lineages (such as ACs, BCs, and Rods) that are naturally partitioned into multiple distinct sub‐clusters by unsupervised methods, we evaluated their aggregated cluster purity across the same parameter setting. For each parameter combination, every resulting unsupervised cluster was assigned to its dominant reference cell lineage using a majority‐voting strategy. The purity of each cluster was then calculated as the proportion of cells belonging to its dominant lineage relative to the total number of cells in that cluster. Finally, to obtain a lineage‐level metric, we calculated the mean cluster purity for each targeted cell type by averaging the purities of all specific unsupervised clusters assigned to it.

### 4.17 Benchmarking of eClassifier Against Existing Annotation Tools

To systematically evaluate the performance of eClassifier, we conducted a head‐to‐head comparison against four widely used cell type annotation tools, including SingleR, CellTypist, ScType, and TransferData in Seurat. To ensure a strictly fair comparison and prevent potential annotation bias driven by the experimental knockout target, the Apoe gene was explicitly excluded from the analyses. Furthermore, all tools utilized the exact same reference and query datasets. SingleR annotation was performed using the SingleR R package (v.2.12.0). We provided the reference expression matrix and labels as input to calculate the Spearman correlation between the query cells and reference transcriptomic profiles using default parameters. For CellTypist (v.1.7.1), a custom reference model was first trained on the reference dataset. The query data were then normalized and log‐transformed, and final predictions were generated using a majority‐voting strategy to refine individual cell labels. To evaluate the marker‐based tool ScType, we constructed a custom marker gene set derived directly from our reference dataset using Seurat. The top 30 significantly up‐regulated genes for each reference cell type were extracted to build the positive marker database. Enrichment scores were subsequently calculated for the scaled query data, and cell types were assigned based on the maximum score. The anchor‐based label transfer was conducted using the standard Seurat pipeline (v.4.3.0). Transfer anchors between the reference and query datasets were identified based on the first 30 principal components, and reference cell type labels were subsequently mapped onto the query dataset.

### 4.18 Cell Location Mapping

To map the transcriptomic profiles of annotated single cells onto the spatial reference, we employed CytoSPACE [19] (v.1.1.0) and TopACT [20] (v.1.1.0). For CytoSPACE mapping, data preprocessing was first performed to ensure alignment accuracy and computational efficiency. For the scRNA‐seq datasets, we filtered out low‐frequency genes expressed in fewer than 1% of total cells to minimize technical noise. For Stereo‐seq spatial data, the dataset was subset based on spatial coordinates to encompass one half of the ocular section, which served as the spatial scaffold. The CytoSPACE algorithm was subsequently executed using four core inputs: the filtered single‐cell raw count expression matrix, the corresponding cell‐type annotation labels, the Stereo‐seq raw spatial expression matrix, and the associated spatial coordinates. This mapping process was conducted utilizing the default parameters to assign individual single cells to their most probable spatial locations on the Stereo‐seq map.

Additionally, we used TopACT to annotate the subcellular spatial transcriptomics data (Stereo‐seq) with cell types inferred from the single‐cell or SeekSpace datasets. To further enhance computational accuracy and efficiency during this process, we provided a coordinate file of spots within the tissue as a spatial mask file to exclude calculations for spots outside the tissue region.

### 4.19 Deconvolution Analysis

To spatially resolve cell type composition across Stereo‐seq bin50 spots, we performed reference‐based deconvolution using the RCTD R package [48]. The RCTD model was trained on the SeekSpace data reference, retaining genes expressed and shared with the Stereo‐seq dataset. Spatial bin50 spots were decomposed into cell type proportions under the RCTD “doublet” mode, which accounts for potential spot‐level cellular mixtures.

### 4.20 Quantification of Cell Radial Distances From the Fitted Center

To analyze the spatial distribution of retinal cells, we employed a geometric circle‐fitting approach. The eye center coordinates were derived through a linear least‐squares optimization, which minimized residuals between observed points and a fitted circular model. For each cell coordinate, we computed its Euclidean distance to this estimated center, generating a quantitative metric of spatial dispersion.

### 4.21 RNAscope Image Analysis

Input image is processed by gaussian difference to detect RNAscope signal. The image is processed with sigma = 1 as Gaussian A and sigma = 4 as Gaussian B, and the signal is defined as GaussianA > (GaussianB * 1.5). Then, we filtered the signal by intensity, signals with lower intensity than 0.0625 are discarded. The inner border and outer border are manually marked to calculate the distance of each signal to each border.

### 4.22 DAPI Image Analysis

Input image is processed by gaussian difference to segment the nucleus. The image is processed with sigma = 1.5 as Gaussian A and sigma = 5 as Gaussian B, and the signal is defined as GaussianA > (GaussianB * 1.0375). Then, we polished the result by binary opening to get nucleus count.

### 4.23 Statistical Analysis

Statistical analysis was performed in R Studio (R version 4.3.0), and graphs were generated using the ggplot2 (v.3.4.3) R package. The number of samples and specific statistical tests performed were described in the figures or figure legends. The statistical significance of data was denoted on graphs by using asterisks (*P‐value < 0.05, **P‐value < 0.01, ***P‐value < 0.001) or NS (not significant).

## Author Contributions

C.D., Y.M.L., Y.Z., and X.X. conceived the study. C.D., S.P.L., Y.J.W. and Y.Y.Q. performed the bioinformatics analysis. Y.M.L., Z.Y.L., Y.D.W, and T.C. performed the experiments. B.Y.L. and Y.L. contributed important information. C.D., Y.M.L., Y.Z., and X.X. wrote the manuscript.

## Conflicts of Interest

None of the authors have a conflict of interest to disclose.

## Code Availability

The source code has been deposited in GitHub (https://github.com/zhouyulab/ASCAL).

## Supporting information




**Supporting File 1**: advs75857‐sup‐0001‐SuppMat.pdf.

**Supporting File 2**: advs75857‐sup‐0002‐Data.zip.

## Data Availability

The scRNA‐seq and spatial transcriptomics raw data generated in this study are deposited in the Genome Sequence Archive (GSA) in BIG Data Center, Beijing Institute of Genomics (BIG), Chinese Academy of Sciences (https://ngdc.cncb.ac.cn/gsa/), under the accession code CRA024710.
