# Supplementary material for: Single‐Cell Annotation and Localization via Integrating Spatial Transcriptomics Maps the Mouse Ocular Atlas and RAO Dynamics
Source: Adv Sci (Weinh). 2026 Jun 1:e75857. Online ahead of print. doi: 10.1002/advs.75857 (PMC13335773; doi:10.1002/advs.75857)
Supplement: Supplementary file 1 — Supporting File 1: advs75857‐sup‐0001‐SuppMat.pdf. [file ADVS-9999-e75857-s001.pdf]

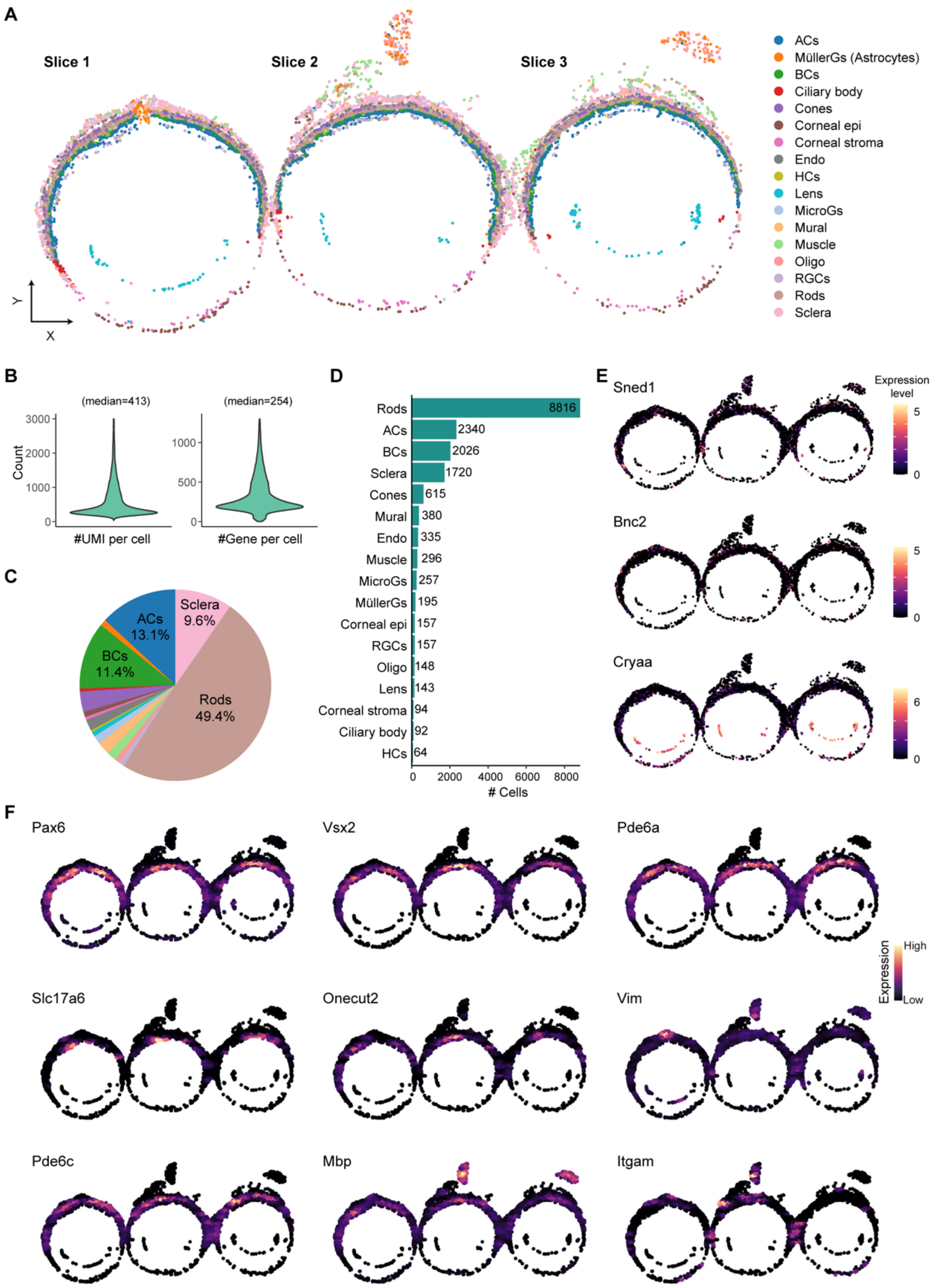

Supplementary Figure 1: Quality control and detailed information of SeekSpace data. (**A**) Tissue-space coordinate plots showing annotated cell types in all three slices. (**B**) Violin plots showing the distributions of UMI (left) and gene (right) counts per cell in SeekSpace data. (**C**) Pie plot showing the major cell percentages in SeekSpace data. Color annotations correspond to those in A. (**D**) Bar plot showing cell number statistics in SeekSpace data. (**E**) Tissue-space coordinate plots visualizing expression patterns of

non-retinal cell-type-specific marker genes. Color gradients indicating gene expression levels. (**F**) Tissue-space coordinate plots visualizing expression patterns of retinal cell-type-specific marker genes. Color gradients indicating gene expression density distributions.

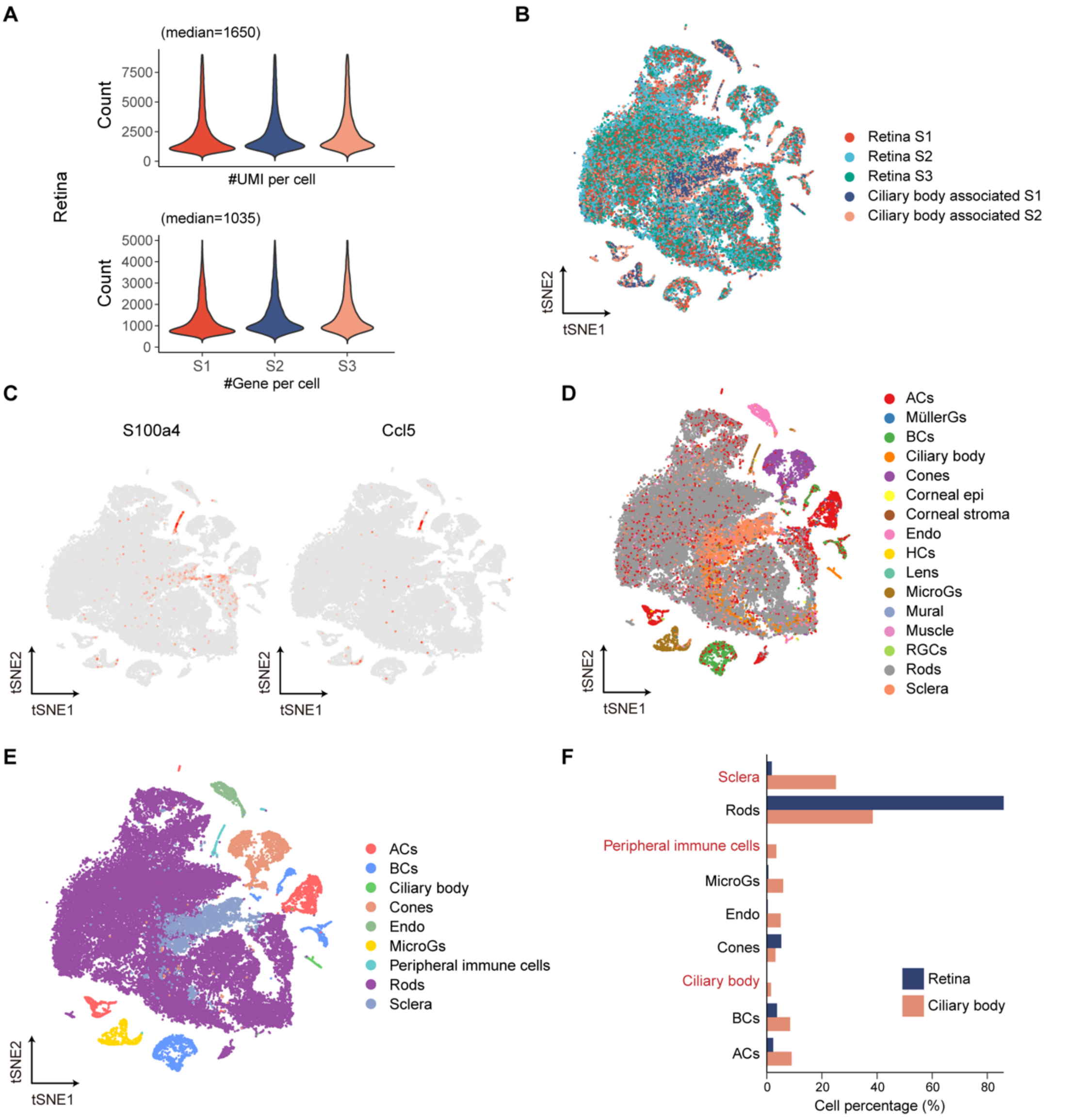

Supplementary Figure 2: Quality control and detailed information of mouse ciliary body and retina scRNA-seq. (**A**) Violin plots showing the distributions of UMI (top) and gene (bottom) counts per cell in scRNA-seq of three retinal samples. (**B**) tSNE projection showing the batch effect-removed cell distributions colored by sample. (**C**) tSNE projection showing expression patterns of peripheral immune cells' marker genes (*S100a4* and *Ccl5*). (**D**) tSNE projection of eClassifier-predicted cell type annotation. (**E**) tSNE projection of cell type annotation adjusted based on unsupervised clustering. (**F**) Bar plot showing the percentage of cell types in the ciliary body and retina samples, respectively.

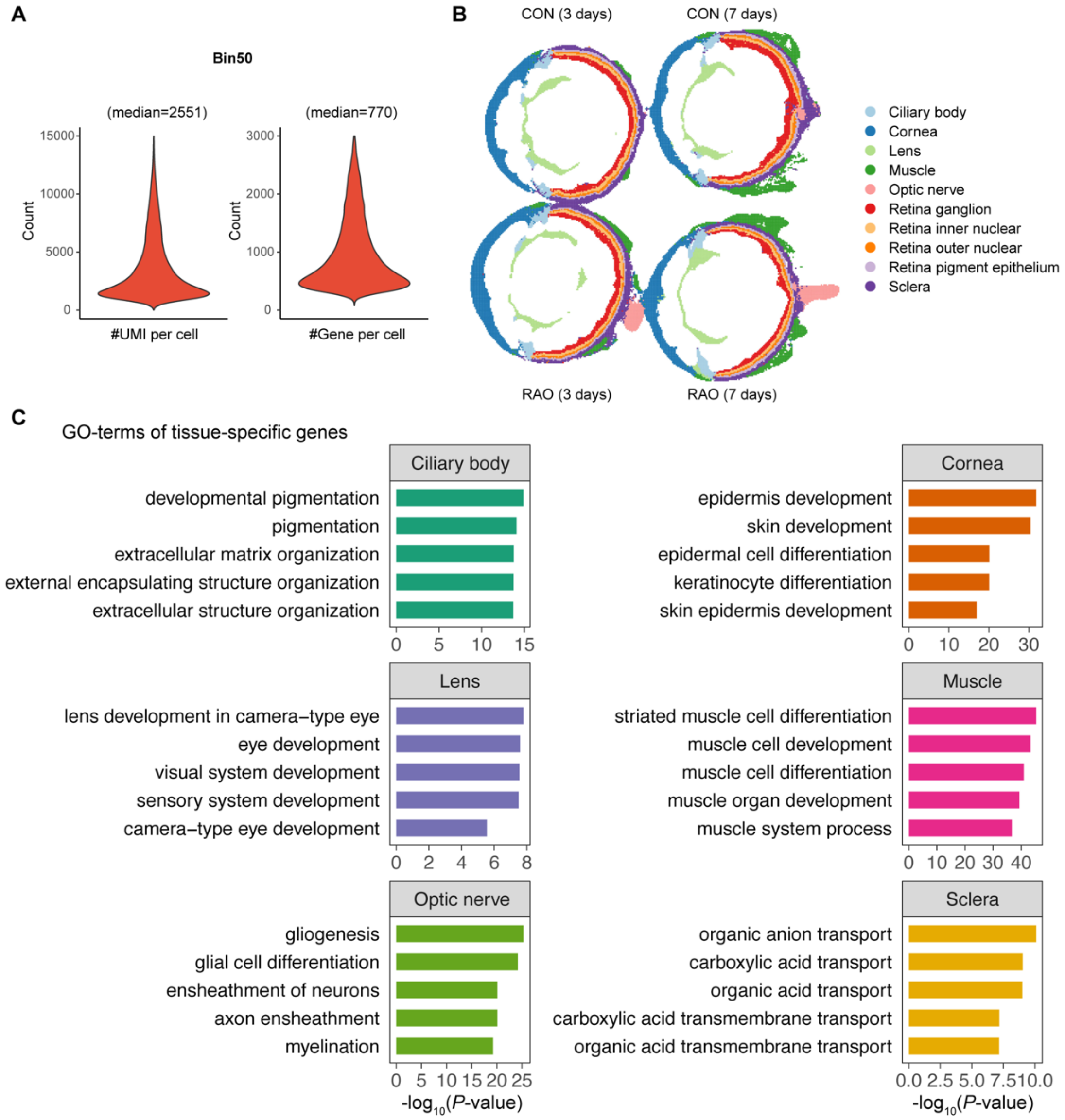

Supplementary Figure 3: Quality control of Stereo-seq and enriched GO-terms of marker genes in non-retina regions. (**A**) Violin plots showing the distributions of UMI (left) and gene (right) counts in Stereo-seq at bin50 resolution. (**B**) Tissue-space coordinate plots showing annotated cell types in all four slices at bin50 resolution. (**C**) Enriched GO-terms (BP) of tissue-specific genes in different non-retina regions.

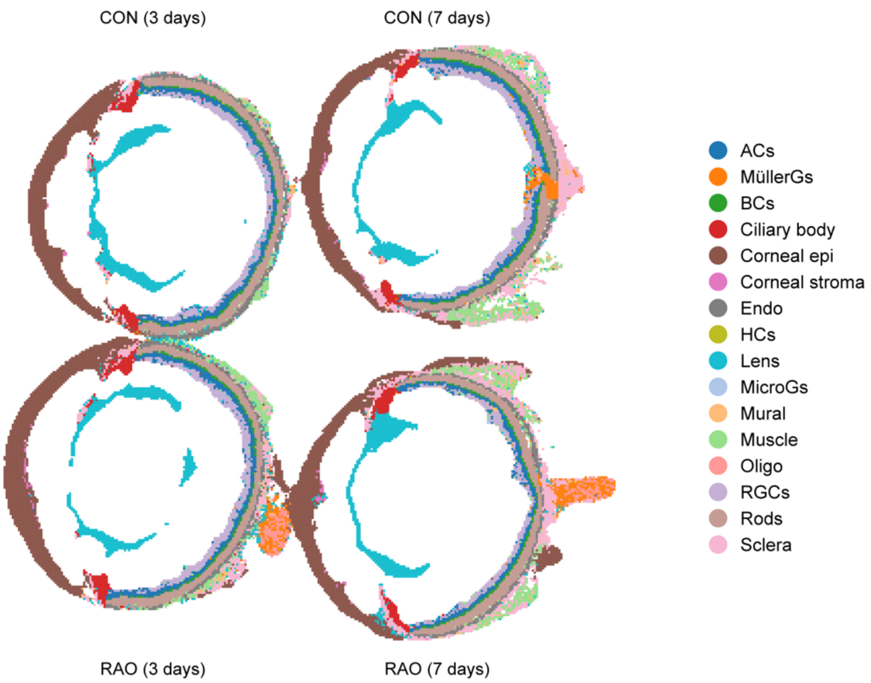

Supplementary Figure 4: Deconvoluted major cell types of Stereo-seq data at bin50 resolution. Tissue-space coordinate plots showing cell-type deconvolution results of Stereo-seq (bin50 resolution) based on SeekSpace data.

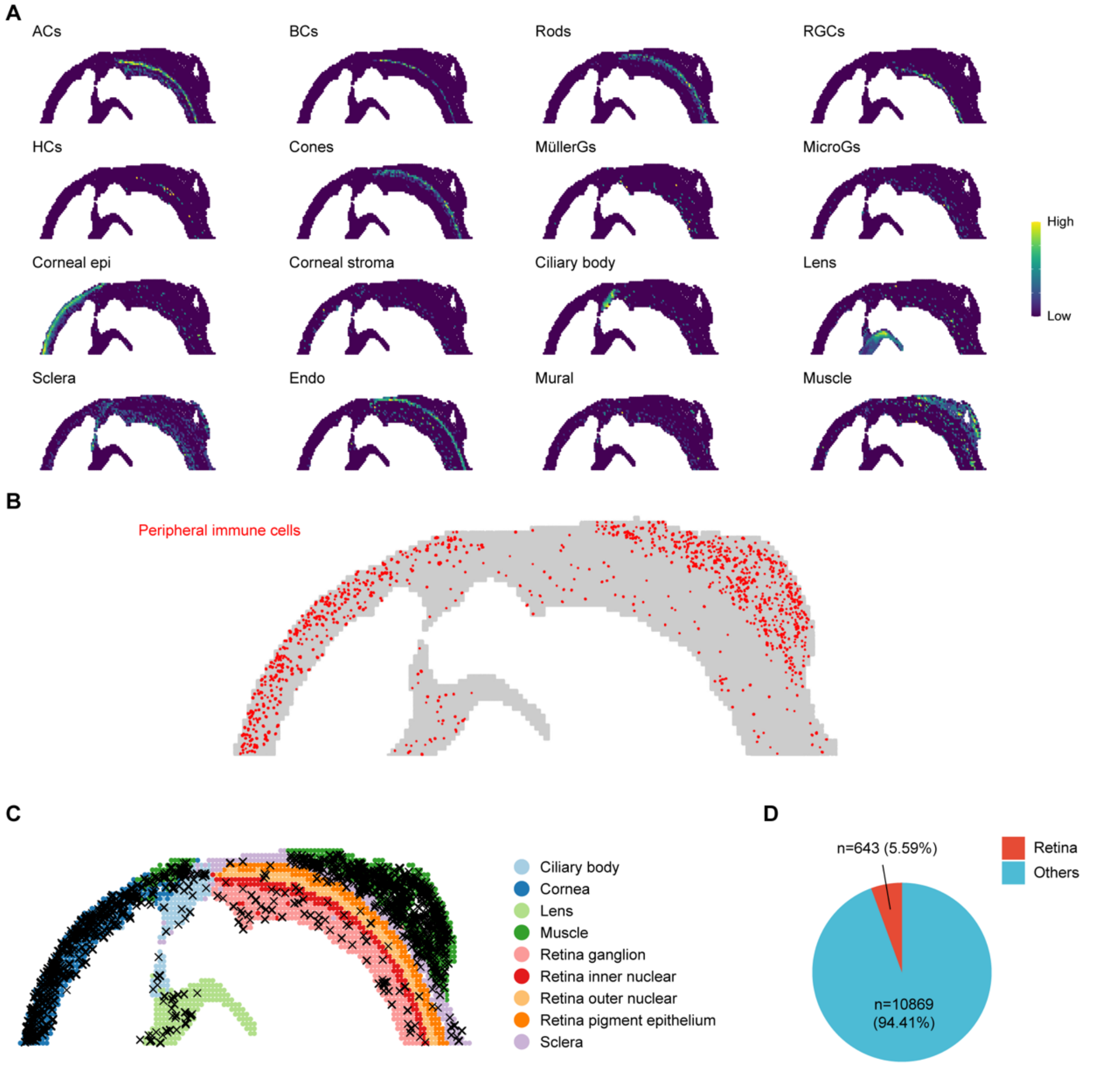

Supplementary Figure 5: Reconstruction of single-cell spatial localization based on Stereo-seq. (**A**) Heatmaps showing SeekSpace data aligning to spatial locations in Stereo-seq at bin50 resolution using CytoSPACE. (**B**) Predicted spatial localization for peripheral immune cells using TopACT based on Stereo-seq at subcellular resolution, with red dots indicating predicted positions with a threshold of 0.6. (**C**) Spatial locations of TopACT-predicted peripheral immune cells (black crosses) within Stereo-seq annotated clusters at bin50 resolution. (**D**) Pie plot showing the distribution of TopACT-predicted peripheral immune cells belonging to retina or not.

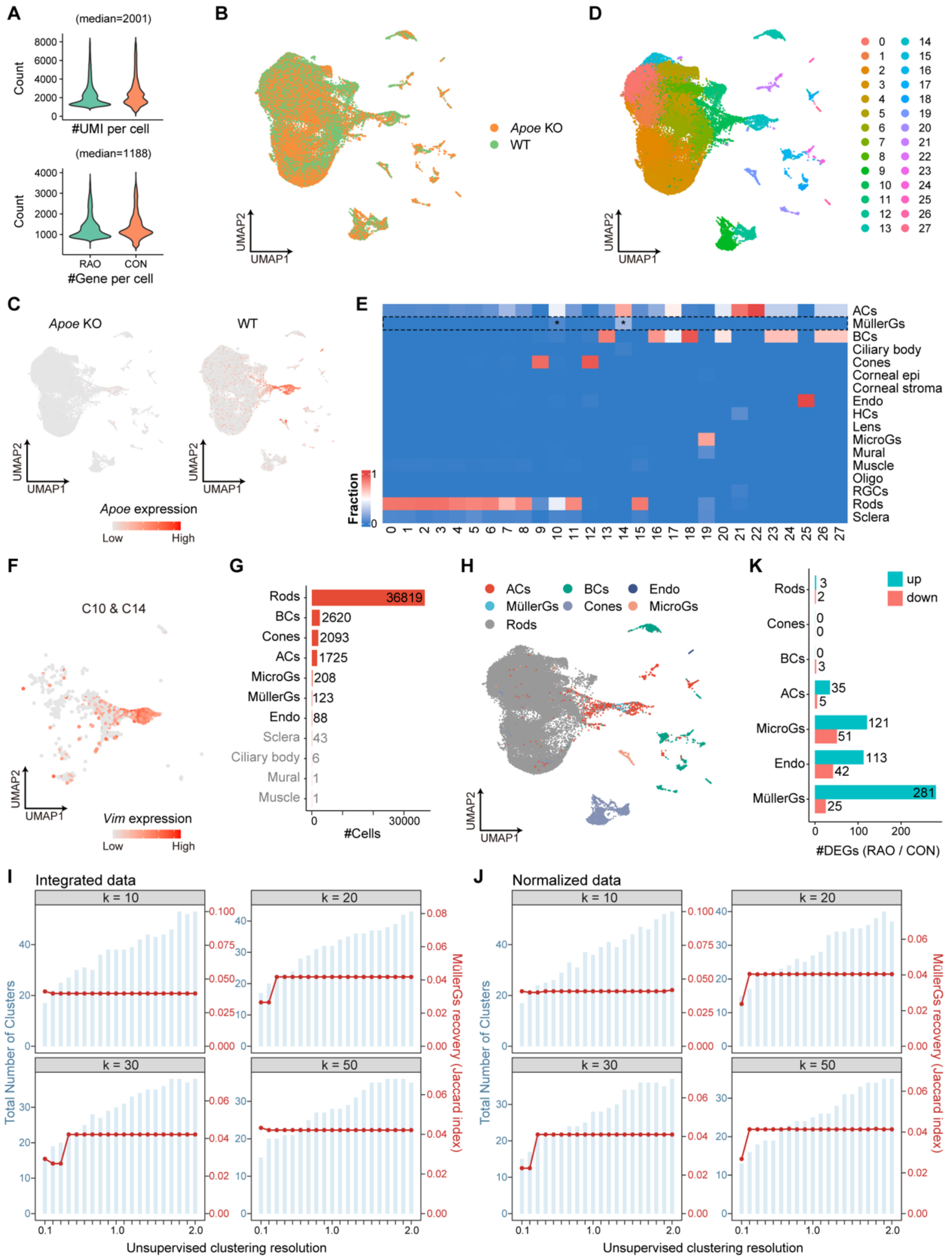

Supplementary Figure 6: Quality control and detailed information of *Apoe* KO mouse retina scRNA-seq data. (**A**) Violin plots showing the distributions of UMI (top) and gene (bottom) counts per cell in *Apoe* KO mouse retina scRNA-seq data. (**B**) UMAP projection showing the batch effect-removed cell distributions colored by genotype. (**C**) UMAP projection showing the expression pattern of *Apoe* in *Apoe* KO and WT samples, respectively. (**D**) UMAP projection of clusters from unsupervised clustering. (**E**) Heatmap showing the eClassifier-predicted cell type fractions in each cluster (0-27) from unsupervised clustering. (**F**) UMAP projection showing the

expression pattern of *Vim* in cells belonging to C10 and C14 clusters. (**G**) Bar plot showing the cell numbers of annotated cell types in *Apoe* KO mouse retina scRNA-seq data, with those containing fewer than 50 cells labelled in gray. (**H**) UMAP projection of annotated cell types of merged data. (**I-J**) The performance of standard unsupervised clustering was evaluated using the integrated data (I) and the normalized data (J). Light blue bars (left y-axis) indicate the total number of distinct clusters generated at each specified resolution (x-axis). The red lines (right y-axis) represent the Jaccard index, which measures the concordance between the Müller glia population identified by eClassifier and the most overlapping cluster generated by the unsupervised approach. (**K**) Bar plot showing the number of differentially expressed genes (DEGs) between RAO and CON by cell type.

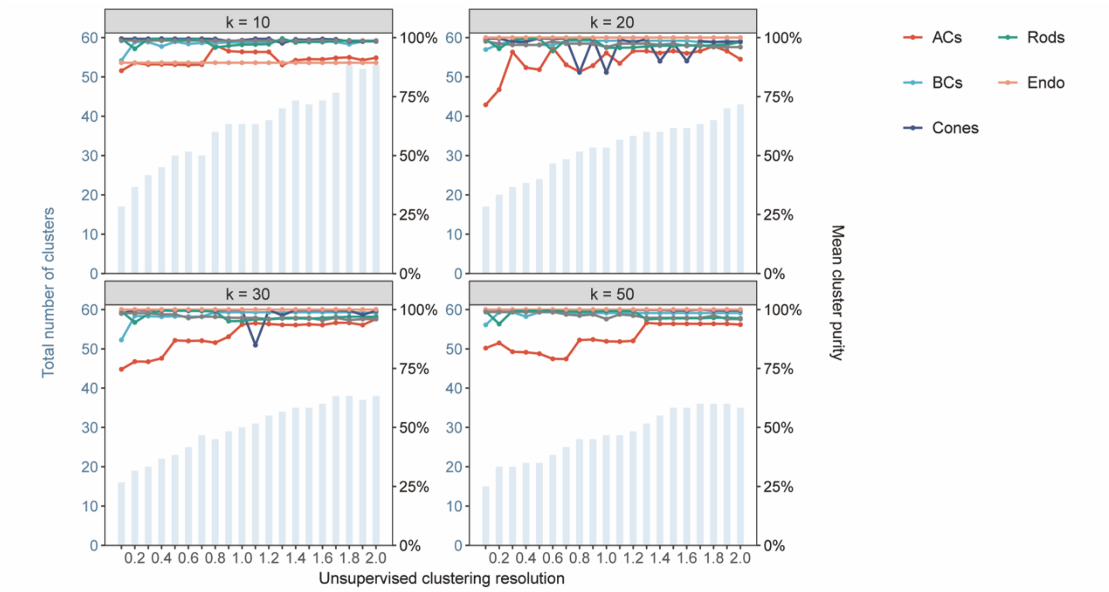

Supplementary Figure 7: Quantitative evaluation of unsupervised cluster purity for highly heterogeneous cell lineages annotated by eClassifier. The performance of unsupervised clustering was evaluated using the integrated data across four neighborhood sizes (k = 10, 20, 30, and 50). Light blue bars (left y-axis) indicate the total number of distinct clusters generated at each specified resolution (x-axis). The colored lines (right y-axis) represent the mean cluster purity for five retinal cell types (ACs, BCs, Rods, Cones, and Endo) annotated by eClassifer.

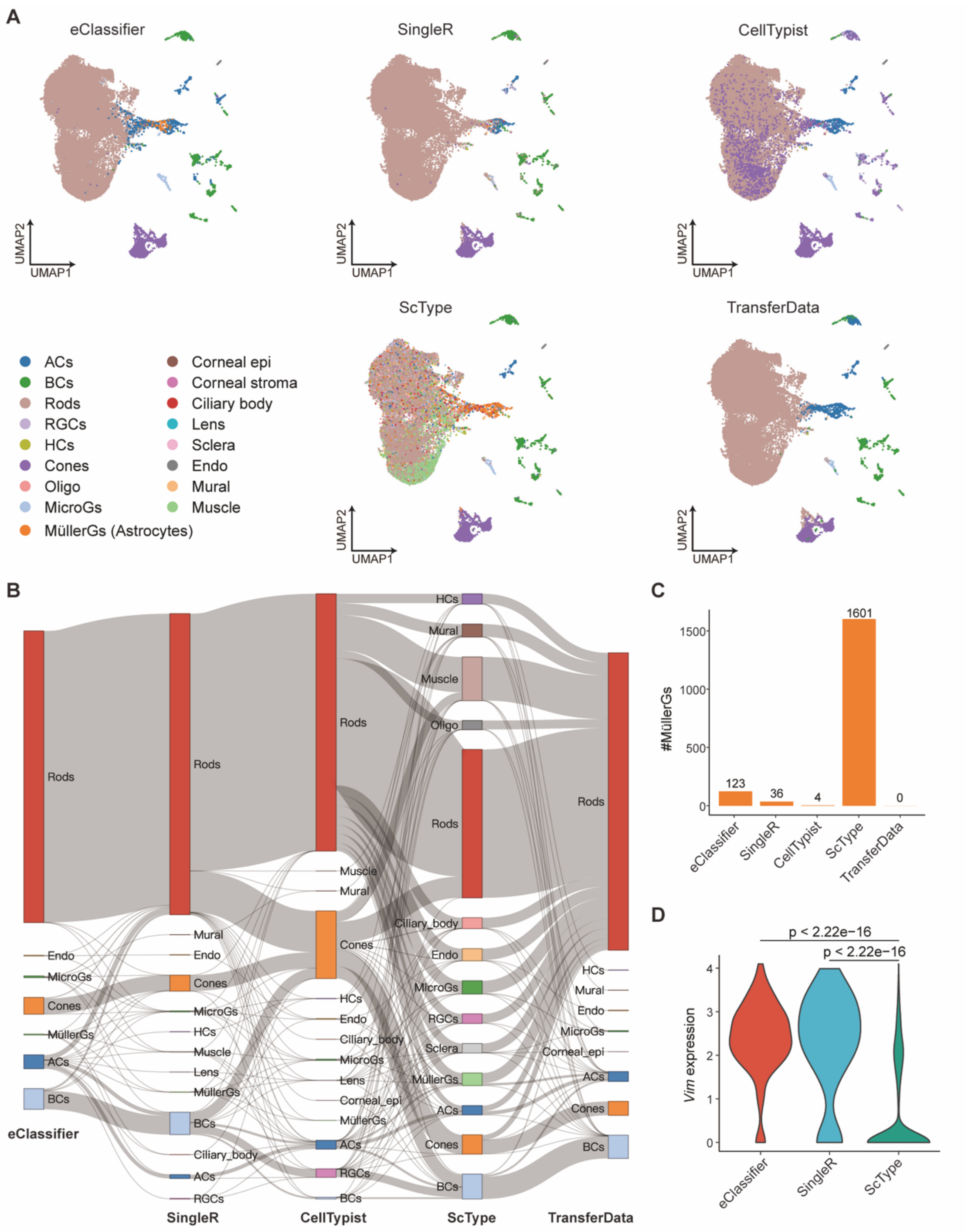

Supplementary Figure 8: Performance benchmarking of eClassifier against alternative cell type annotation methods. (**A**) UMAP visualization of retinal cell clusters annotated by eClassifier and four representative tools (SingleR, CellTypist, ScType, and TransferData). (**B**) Sankey diagram illustrating the classification flow across different annotation pipelines. (**C**) Bar plot showing the predicted cell counts of Müller glia (MüllerGs) across methods. (**D**) Violin plot depicting the expression levels of *Vim* (marker gene of MüllerGs) in clusters annotated as MüllerGs by different methods.

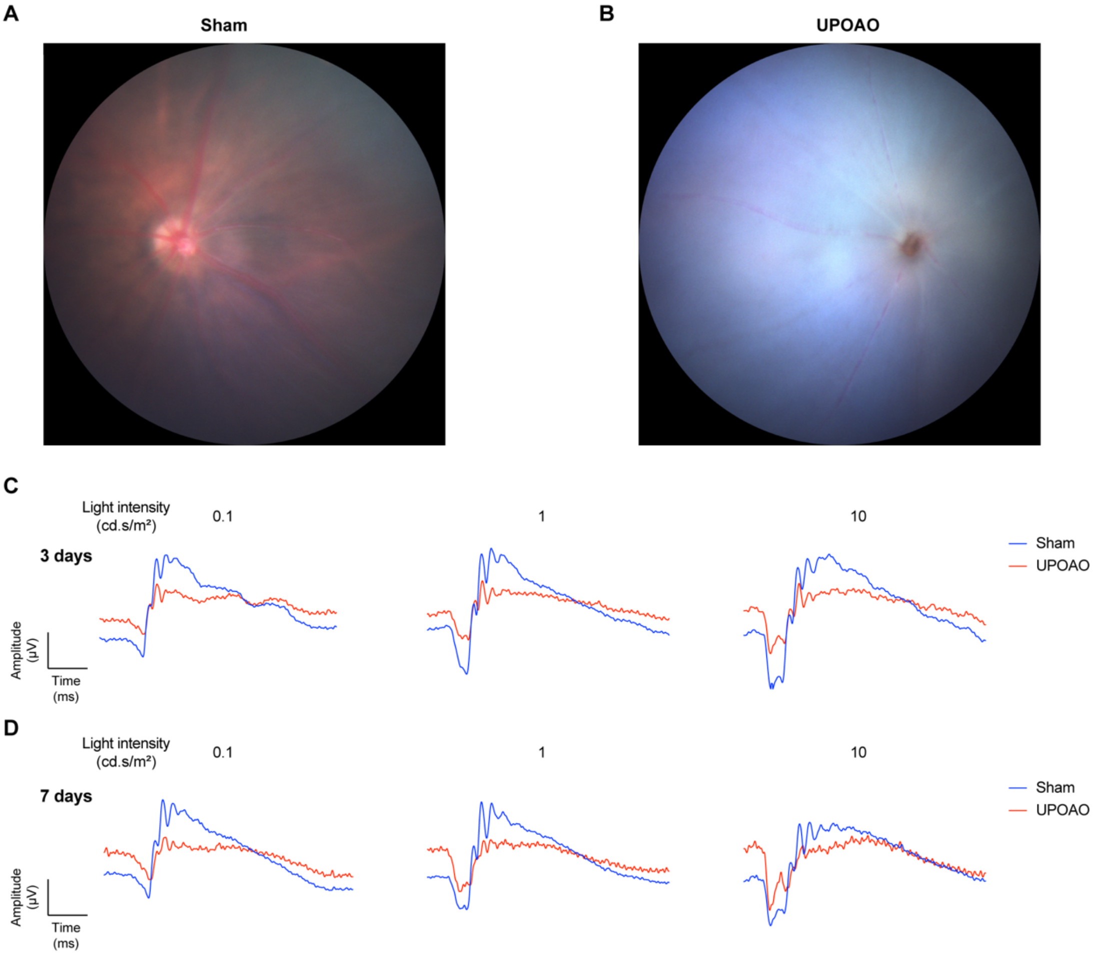

Supplementary Figure 9: Validation of the RAO model. (**A**) Fundus image of the Sham group, showing normal fundus vessels and optic disc appearance. (**B**) Fundus image of the UPOAO model during embolus insertion. (**C-D**) The electroretinogram (ERG) results of the UPOAO operation at 3 days (C) and 7 days (D) after reperfusion, recorded under different light intensities (0.1, 1, and 10 cd.s/m²). The Sham and UPOAO groups are colored in blue and red, respectively.

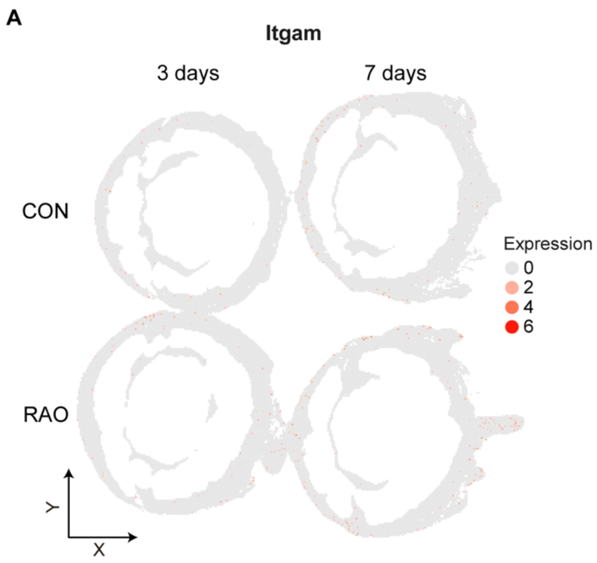

Supplementary Figure 10: *Itgam* expression pattern. (**A**) Space coordinate plot showing *Itgam* expression pattern in the four Stereo-seq slices.

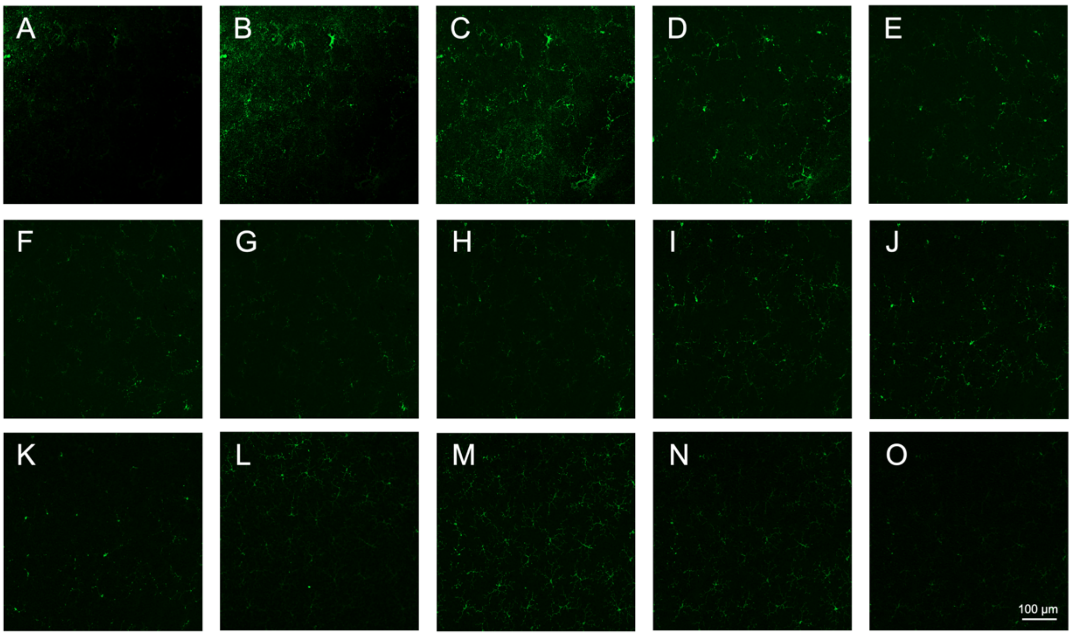

Supplementary Figure 11: Representative z-stack confocal images of IBA1-labeled retinal microglia in CON (SHAM) mice. Fifteen representative z-slices of IBA1 immunofluorescence (green) were selected from complete z-stack scans of CON (SHAM) mouse retinas, to span the full retinal thickness.

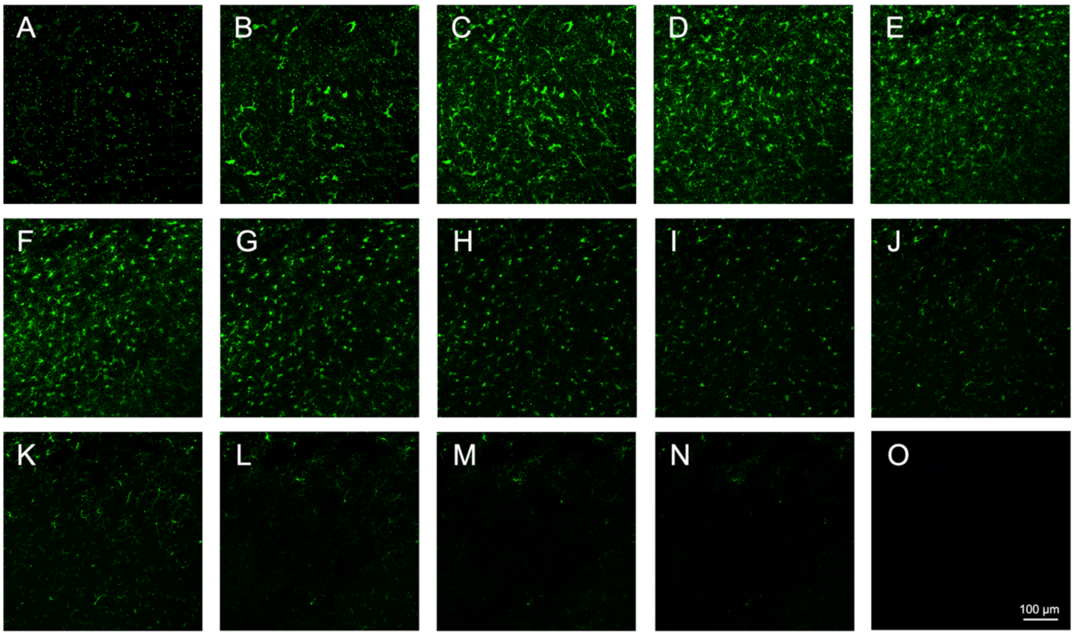

Supplementary Figure 12: Representative z-stack confocal images of IBA1-labeled retinal microglia in RAO (UPOAO) mice. Fifteen representative z-slices of IBA1 immunofluorescence (green) were selected from complete z-stack scans of RAO (UPOAO) mouse retinas, to span the full retinal thickness.

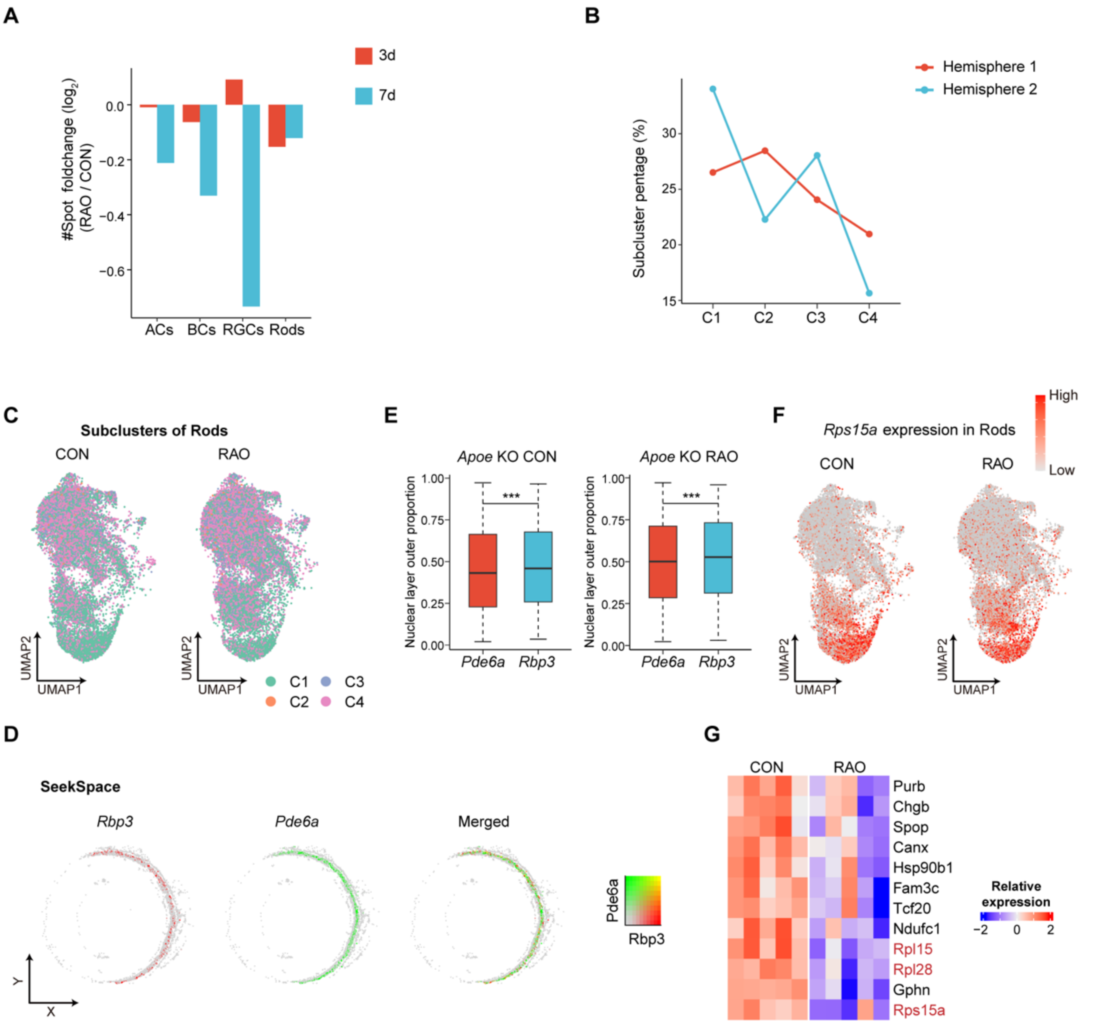

Supplementary Figure 13: Cellular alterations between RAO and control (CON). (**A**) Bar plot showing the spot number changes between RAO and CON for each deconvoluted cell type. (**B**) Line plot showing the percentage of each rod subcluster in the two hemispheres of the mouse eye. (**C**) UMAP projections of eClassifier-predicted Rod subcluster annotation. (**D**) Space coordinate plots showing *Rbp3* and *Pde6a* expression patterns in the same SeekSpace slice. (**E**) Box plots showing the outer proportion of *Pde6a* and *Rbp3* signals in control (CON) (left panel) and RAO (right panel) eye sections of *Apoe* KO mice. The *P*-values were calculated by the Wilcoxon test. (**F**) UMAP projections showing the expression pattern of *Rps15a* in Rods under CON and RAO, respectively. (**G**) Heatmap showing the relative expression of C1 subcluster representative marker genes among Rods in retinal bulk RNA-seq from RAO and CON samples (data from Wang et al. *eLife* 2024).

Description of Additional Supplementary Files

File Name: Supplementary Data 1

Description: List of tissue-specific genes for each non-retina region identified in Stereo-seq bin50 analysis.

File Name: Supplementary Data 2

Description: List of Müller glia up-regulated genes under RAO treatment from *Apoe* KO mouse retina scRNA-seq data.
